# Positive charges promote the recognition of proteins by the chaperone SlyD from *Escherichia coli*

**DOI:** 10.1371/journal.pone.0305823

**Published:** 2024-06-25

**Authors:** Daniel Lindemeier, Wenke Graubner, Denise Mehner-Breitfeld, Miroslav Malešević, Thomas Brüser

**Affiliations:** 1 Institute of Microbiology, Leibniz Universität Hannover, Hanover, Germany; 2 Institute of Biochemistry and Biotechnology, Martin-Luther-University Halle-Wittenberg, Halle, Germany; University of Delhi, INDIA

## Abstract

SlyD is a widely-occurring prokaryotic FKBP-family prolyl isomerase with an additional chaperone domain. Often, such as in *Escherichia coli*, a third domain is found at its C-terminus that binds nickel and provides it for nickel-enzyme biogenesis. SlyD has been found to bind signal peptides of proteins that are translocated by the Tat pathway, a system for the transport of folded proteins across membranes. Using peptide arrays to analyze these signal peptide interactions, we found that SlyD interacted only with positively charged peptides, with a preference for arginines over lysines, and large hydrophobic residues enhanced binding. Especially a twin-arginine motif was recognized, a pair of highly conserved arginines adjacent to a stretch of hydrophobic residues. Using isothermal titration calorimetry (ITC) with purified SlyD and a signal peptide-containing model Tat substrate, we could show that the wild type twin-arginine signal peptide was bound with higher affinity than an RR>KK mutated variant, confirming that positive charges are recognized by SlyD, with a preference of arginines over lysines. The specific role of negative charges of the chaperone domain surface and of hydrophobic residues in the chaperone active site was further analyzed by ITC of mutated SlyD variants. Our data show that the supposed key hydrophobic residues of the active site are indeed crucial for binding, and that binding is influenced by negative charges on the chaperone domain. Recognition of positive charges is likely achieved by a large negatively charged surface region of the chaperone domain, which is highly conserved although individual positions are variable.

## Introduction

**Fig 1:**
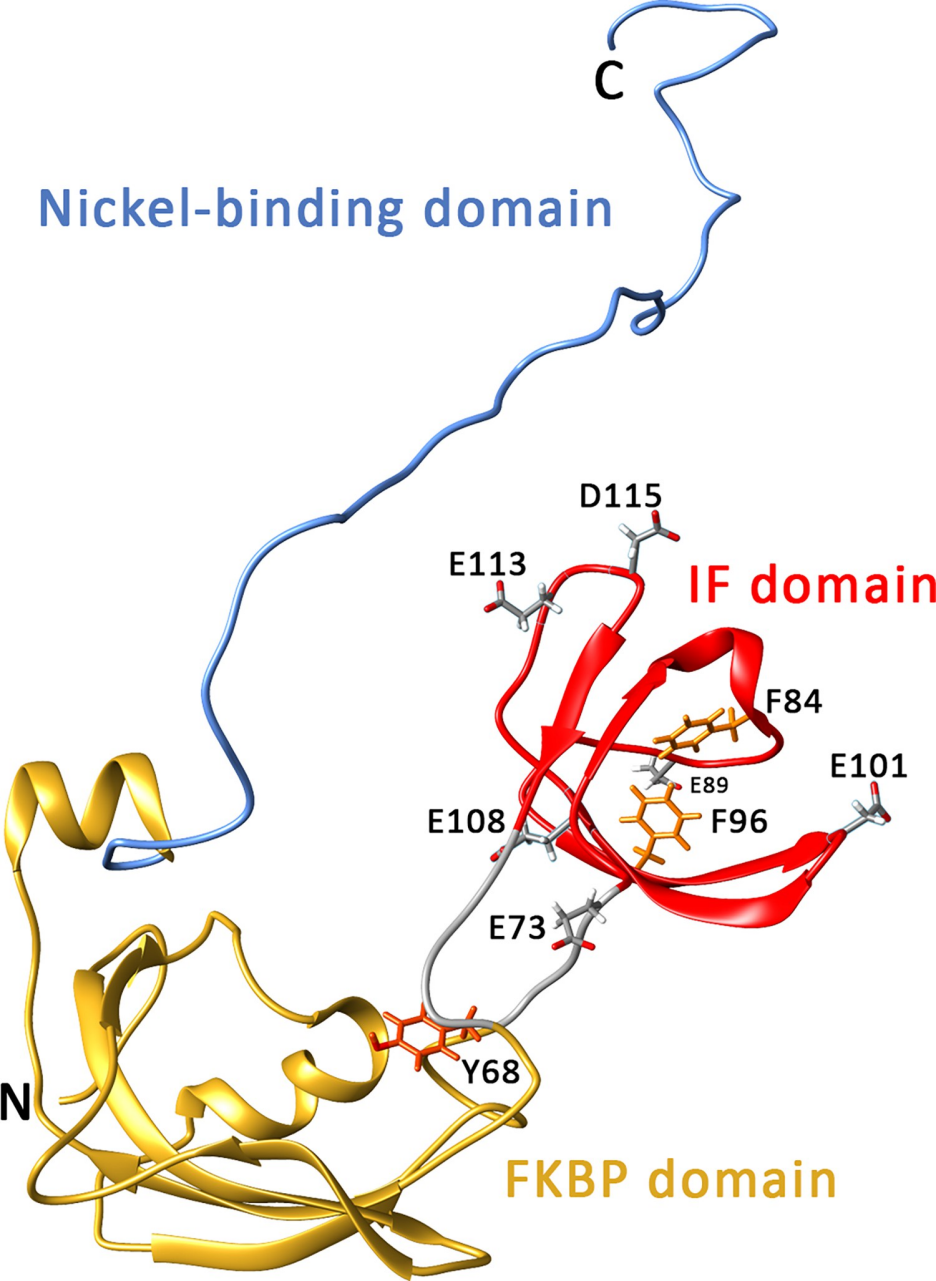
Domain structure of SlyD. Figure based on PDB entry 2KFW, which is a solution NMR structure of full-length SlyD from *E*. *coli*. Linker region in grey. Residues mutated in this study are indicated.

*Escherichia coli* SlyD is a protein with general chaperone, prolyl isomerase and nickel-chelating domains (Fig 1, [1]). It belongs to the FK506-binding protein (FKBPs) prolyl isomerase family of pro- and eukaryotes [2]. The chaperone domain is **i**nserted in a **f**lap of the prolyl isomerase domain and is therefore termed IF domain [3]. The *slyD* gene has been originally discovered as locus required for cell lysis by phage ΦX174, and it has later been shown that the phage lysis protein E is chaperoned by SlyD, which prevents degradation before E reaches its target at the inner membrane [4–8]. The C-terminal nickel-chelating domain provides nickel for hydrogenases [9–12]. This domain is typically found in γ- and ϵ-proteobacteria, and sometimes it occurs also in other phyla (S1 Fig). However, it is absent in many species, indicating that SlyD has functions beyond nickel storage, and these functions rely on the chaperone and/or prolyl isomerase domains. Tat substrate folding or targeting might be such a function, as SlyD has been found to bind the Tat signal peptides of HiPIP, AmiA, EfeB (= YcdB), and CueO [13]. Tat substrates are proteins that fold inside the prokaryotic cytoplasm before being translocated across the cytoplasmic membrane [14]. In agreement with a role in Tat transport, SlyD from *Campylobacter jejuni* has been recently shown to bind the Tat signal peptide of CueO also in this organism, where it contributes to CueO transport into the periplasm and thereby to copper resistance [15]. Other reported SlyD functions that relate to modulations of pathway efficiencies by direct interactions include the regulation of DNA-binding ability of the ferric uptake regulator Fur (in cooperation with YdiV), as observed in *E*. *coli* [16], and the posttranslational modulation of the activity of the Nickel transporter Niu in *Helicobacter pylori* [17]. The chaperone domain was more important in the latter case, whereas the prolyl isomerase was required in the first case.

The flexibly connected domains that confer diverse functions to SlyD make this chaperone a versatile protein that is highly interesting for biochemical and biophysical studies. The chaperone and prolyl isomerase activities of SlyD from *E*. *coli* and *Thermus thermophilus* have been extensively studied in conjunction with structural analyses [18–22]. It has been shown that the recombinant insertion of the *E*. *coli* SlyD IF domain into human prolyl isomerase FKBP12 enhanced prolyl isomerase activity with protein substrates ca. 200-fold [23, 24]. The removal of the IF domain from SlyD abolished its prolyl isomerase activity with protein substrates, whereas the activity with a small peptide substrate remained unaltered [23]. With longer peptides, it has been shown that binding to the IF domain clearly enhanced prolyl isomerase activity at the FKBP domain, and the IF domain influenced the orientation of peptide-binding to the prolyl isomerase active site [25]. A structural cross-talk between the two domains has been demonstrated for *E*. *coli* and *T*. *thermophilus* SlyD [26]. SlyD was found to catalyze prolyl cis/trans isomerization much faster than conformational folding, and the generation of incorrect trans isomers by SlyD therefore can retard folding processes [27].

Binding sites of the chaperone and prolyl isomerase domains of *E*. *coli* SlyD have been initially mapped by ^15^N HSQC NMR spectroscopy, which identified residues whose amide proton signals were shifting in response to substrate-binding [3]. First direct visualization of peptide-binding was provided by studies on *T*. *thermophilus* SlyD, whose crystal structures have been solved with bound peptide substrates [28]. In the latter study, also mutagenesis studies have been carried out to identify functionally important residues. The mutated residues included six positions in the FKBP domain (of these, Y63 is a highly conserved residue of the prolyl isomerase active site and positioned at the border to the first inter-domain loop; it corresponds to Y68 of *E*. *coli* SlyD), one in the inter-domain loop, and four residues in the IF domain [28]. So far, none of the key hydrophobic residues of the IF domain active site were mutated, and mutations in the surrounding of the hydrophobic pocket were analyzed that had only little effect on substrate affinities. For efficient binding, two hydrophobic residues of bound substrates can be enclosed by the hydrophobic residues of the chaperone active site [19]. In crystal structures, the polar regions of bound peptides were detected at various different positions, indicating significant variability of potential interactions with polar or charged regions of the peptides [19]. It has to be kept in mind that the crystals were obtained under high salt conditions that may interrupt ionic interactions with the IF domain surface and that promote hydrophobic effects. The IF domain can bind peptides by donor strand complementation, a stabilization of a β-barrel structure by a β-strand coming from the bound substrate [19]. SlyD can also bind α-helices, which requires a distinct binding mode, demonstrating a remarkable heterogeneity of SlyD substrate interactions [8].

In our efforts to characterize Tat signal peptide recognition by SlyD, we performed peptide scans, which showed that positively charged residues in peptides are required for recognition by SlyD. This prompted us to further analyze the role of these interactions in more detail. Our data demonstrate a so far unrecognized role of charge interactions between the bound substrates and multiple possible positions on the surface of the IF domain. This mechanism appears to be conserved in SlyD and is reminiscent to DnaK, another chaperone that binds hydrophobic residues adjacent to positive charges.

## Materials and methods

### Strains and growth conditions

*E*. *coli* DH5α (Invitrogen) and XL1-Blue (Agilent) were used for cloning, *E*. *coli* BL21(DE3) (Agilent) and its derivative BL21(DE3) Δ*slyD* were used for inclusion body production for HiPIP and SlyD, respectively. The Keio collection strains BW25113, BW25113 Δ*slyD*, BW25113 Δ*dnaK* [29] as well as *E*. *coli* MC4100 [30] or its derivatives MC4100 Δ*cueO* or MC4100 Δ*slyD* were used for physiological and biochemical experiments, as indicated. The bacteria were aerobically grown at 37°C in LB medium (1% tryptone, 1% NaCl, 0.5 yeast extract) supplemented with required antibiotics (100 μg/mL ampicillin, 25 μg/mL chloramphenicol, 15 μg/mL kanamycin).

### Plasmids and genetic methods

The Keio collection strains BW25113 Δ*cueO* and BW25113 Δ*slyD* [29] were used to construct the strains MC4100 Δ*cueO*, MC4100 Δ*slyD*, and BL21(DE3) Δ*slyD*, respectively, by P1 transduction according to standard protocols [31]. The recipient clones were selected and purified on LB agar plates containing kanamycin (50 μg/mL). The position of the kanamycin cassette was confirmed by colony PCR using the primers k2, kt, *slyD*:kan:-F, *slyD*:kan:-R, *cueO*-test-F and *cueO*-test-R (see Table 1). For recombinant production of HiPIP or C-terminally His-tagged SlyD inclusion bodies, the expression plasmids pEXH5 or its derivative pEX-*slyD*_*H6*_ were used, respectively [32]. Single amino acid exchanges in the *slyD* gene were introduced by QuikChange mutagenesis (Stratagene) of pEX-*slyD*_*H6*_ (see Table 1 for list of primers). For production of soluble SlyD, C-terminally tagged with a Strep-tag II, the vector pBW-*slyD*_*strep*_ was used [13]. All constructs and mutations were confirmed by sequencing.

**Table 1:** Primers used in this study^1^.

QuikChange Mutagenesis Primers^2^	Sequence (5’>3’)
*hip*-QQ-F	CGATAAGCCAATCAGCAAGAGCCAACAGGACGCTGTCAAAGTGATGCTGG
*slyD*_*H6*_-Y68S-F	GTTGGCGCGAACGACGCTAGCGGTCAGTACGACGAAAAC
*slyD*_*H6*_-E73A-F	CGGTCAGTACGACGCAAACCTGGTGCAAC
*slyD*_*H6*_-F84A-F	CAACGTGTTCCTAAAGACGTAGCTATGGGCGTTGATGAACTGC
*slyD*_*H6*_-F84N-F	CAACGTGTTCCTAAAGACGTAAATATGGGCGTTGATGAACTGC
*slyD*_*H6*_-E89A-F	GTATTTATGGGCGTTGATGCACTGCAGGTAGGTATGCGT
*slyD*_*H6*_-F96A-F	CAGGTAGGTATGCGTGCCCTGGCTGAAACCG
*slyD*_*H6*_-F96G-F	GCAGGTAGGTATGCGTGGCCTGGCTGAAACCGACC
*slyD*_*H6*_-F96N-F	CTGCAGGTAGGTATGCGTAACCTGGCTGAAACCGACC
*slyD*_*H6*_-D101A-F	CTGGCTGAAACCGCCCAGGGTCCGGTAC
*slyD*_*H6*_-E108A-F	GGGTCCGGTACCGGTTGCAATCACTGCGGTTGAAG
*slyD*_*H6*_-E113A-F	GAAATCACTGCGGTTGCAGACGATCACGTCGTG
*slyD*_*H6*_-D115A-F	CACTGCGGTTGAAGACGCTCACGTCGTGGTTGATG
Other primers	Sequence (5’>3’)
*cueO*-test-F	GACGGCCATCAGGCTGCCGAATAAC
*cueO*-test-R	CTATATTGTGGCTTATGCGCTGCCGGATG
k2	CGGTGCCCTGAATGAACTGC
kt	CGGCCACAGTCGATGAATCC
*slyD*:kan:-F	GCCACCGCCACATTATTGAG
*slyD*:kan:-R	GTACACGGCTGCAGAATTCC

^1^Primer sequences are given in 5′-3′ direction.

^2^Corresponding reverse primers covered the same sequence.

### Biochemical and biophysical methods

#### Peptide spot arrays

The cellulose-bound peptide scan of all Tat signal peptide sequences from *E*. *coli* and the signal peptide of HiPIP from *Allochromatium vinosum* consisted of 13-mer overlapping peptides shifted by one or two amino acids, anchored via a C-terminal (β-Ala)_2_ spacer on the membrane. The cellulose-bound peptide scan was prepared by the standard automated spot synthesis protocol [33] using a pipetting robot (Abimed GmbH, Langenfeld, Germany). Briefly, two β-Ala residues were used for a Whatman 540 (Whatman, Dassel, Germany) cellulose membrane derivatization and spots definition. In each step, 9-fluorenylmethyloxycarbonyl (Fmoc) protection was removed by a membrane treatment (1x5 min, 1x15 min) with 20% (vol/vol) piperidine solution in *N*, *N*-dimethylformamide (DMF). For coupling steps, Fmoc-protected amino acids, pre-activated as pentafluorophenyl esters, were used (0.3 M solution in *N*-methyl-2-pyrrolidone, spotted 0.3 μl 3 times). The side chains of the amino acids were protected as follows: 2,2,4,6,7-pentamethyldihydrobenzofuran-5-sulfonyl (Arg), trityl (Asn, Gln, His, Cys), tert-butyl (Thr, Tyr, Ser, Asp, Glu), tert-butyloxycarbonyl (Lys, Trp). After coupling, all unmodified amino groups were capped with a mixture of 0.5 M acetic acid anhydride, 0.125 M *N*,*N*-diisopropylethylamine, 0.015 M 1-hydroxy-benzotriazole in DMF. In the final cycle, the Fmoc-deprotection step had been done before the capping step. Thus, N-terminal acetylated peptides were obtained. At the end of the synthesis, the side chain-protecting groups were removed by treatment with 50% (vol/vol) trifluoroacetic acid/dichloromethane. Prior to incubations with ^35^S-methionine-labeled SlyD, the dry membranes were wetted in methanol and then three times washed for 20 min in TBS buffer (31 mM Tris-HCl pH 7.6, 170 mM NaCl, 6.4 mM KCl).

For production of ^35^S-labeled SlyD, strain BW25113 Δ*dnaK* containing the plasmid pBW-*slyD*-*strep* was grown in 100 ml cultures at 30°C in M9 minimal medium [34], supplemented with 0.1% yeast extract, 0.5% glycerol and 100 μg/mL ampicillin. Gene expression was induced by addition of 0.4% rhamnose at OD600 ~0.4, and 0.2 μCi ^35^S methionine was added (20 μl of 10 mCi/ml, MP Biomedicals). Cells were harvested by centrifugation, resuspended in 100 ml 20 mM Tris-HCl pH 8.0, 150 mM NaCl, disintegrated by sonication, cell debris and membranes were removed by centrifugation (20 min 20.000 g, and 90 min 109.000 g, respectively), and the *Strep*-tagged SlyD was purified by StrepTactin affinity chromatography, according to the manufacturer’s protocol (Iba Lifesciences). ^35^S-methionine-labeled SlyD was incubated with the membranes for 2 h at room temperature. Thereafter, the membranes were washed 3 x 5 min in TBS buffer to remove unbound SlyD, then dried and the signals were recorded using phosphorimaging (STORM 840, GE Healthcare). Quantification of the signals was done using ImageJ [35].

#### Protein purifications and protein folding

Preparation of precursor HiPIP was performed as previously described [36]. For ITC analyses of SlyD and its variants, C-terminally hexahistidine-tagged SlyD was refolded from inclusion bodies by a method developed based on a previously published protocol [37]. Briefly, recombinant production of SlyD variants in *E*. *coli* BL21(DE3) Δ*slyD* harboring pEX*slyD*_*H6*_ or its derivatives was induced at an OD_600_ of about 1.2 with 1 mM IPTG. Cells were harvested by centrifugation (20 min 7,000xg) three hours after induction. Pellets were frozen in liquid nitrogen and stored at -20°C. For cell lysis, the pellet was resuspended in chilled 100 mM sodium phosphate (pH 8.0), 6 M guanidinium hydrochloride (GdnHCl) and 10 mM imidazole, and stirred for 2 h on ice. After centrifugation (20 min 20,000xg) and filtration with Filtropur S membranes (pore size 0.2 μm; Sarstedt), the lysate was applied onto PureCube 100 INDIGO Ni-Agarose (Cube Biotech), equilibrated with lysis buffer supplemented with 2.5 mM Tris(2-carboxyethyl)phosphine (TCEP). The first washing step was performed with 13 volumes of 50 mM sodium phosphate (pH 8.0), 6 M GdnHCl, 20 mM imidazole and 2.5 mM TCEP. SlyD refolding on the matrix was induced by washing with 13 volumes of refolding buffer (50 mM sodium phosphate pH 7.8, 100 mM NaCl, 5 mM imidazole, 2.5 mM TCEP, 1 mM PMSF). The refolding buffer was then replaced with 8 volumes of 50 mM sodium phosphate (pH 7.8), 100 mM NaCl and 5 mM imidazole), and the folded protein was eluted with 6 volumes of the same buffer with 250 mM imidazole. Protein concentration measurements were performed with a DeNovix DS-11+ spectrophotometer (DeNovix). The extinction coefficients for SlyD variants were calculated using ProtParam [38]. For HiPIP, the previously determined extinction coefficients were taken [39]. Protein purity and folding were assessed by SDS-PAGE [40], Western-blotting, and circular dichroism spectroscopy, as previously described [41] (S3 Fig).

#### Isothermal titration calorimetry

The interaction studies were performed with a VP-ITC (MicroCal) at 30°C. SlyD and HiPIP were dialyzed in 10 mM potassium phosphate buffer (pH 7.6 or 7.8, as indicated). For titrations with SlyD and its mutated variants, the SlyD concentration in the calorimetric cell was 30 μM. The concentration of the injected signal-peptide-containing HiPIP precursor was 200 μM for the experiments shown in Fig 3 (23 injections of 10 μL, preceded by a 2 μl injection), and 100 μM for the analyses shown in Fig 4 (2x 22 injections of 10 μL, each set of injections preceded by a 2 μl injection). The two sets of injections were combined using the MicroCal Concat ITC software (Malvern Panalytical). As expected for a tag at the C-terminus, the His-tag had no influence on the titrations, as SlyD with a C-terminal Strep-tag II gave the same results (S4 Fig).

#### Bioinformatic analyses

*E*. *coli* SlyD orthologs from diverse bacteria and archaea (listed in S1 Table) were identified using BLASTP [42]. Clustal Omega was used for sequence alignments and positional conservation of residues was visualized by WebLogo [43, 44]. The phylogenetic tree was created using the NGPhylogeny.fr webtool, and further modified using iTOL v6 [45, 46]. Isoelectric point analyses were performed using the Protein Isoelectric Point workflow of the Sequence Manipulation Suite (SMS) [47]. Protein structures and protein surface properties were visualized using Chimera 1.2 [48].

## Results

### SlyD can bind to the beginning and often also to the end of h-regions in Tat signal peptides

**Fig 2:**
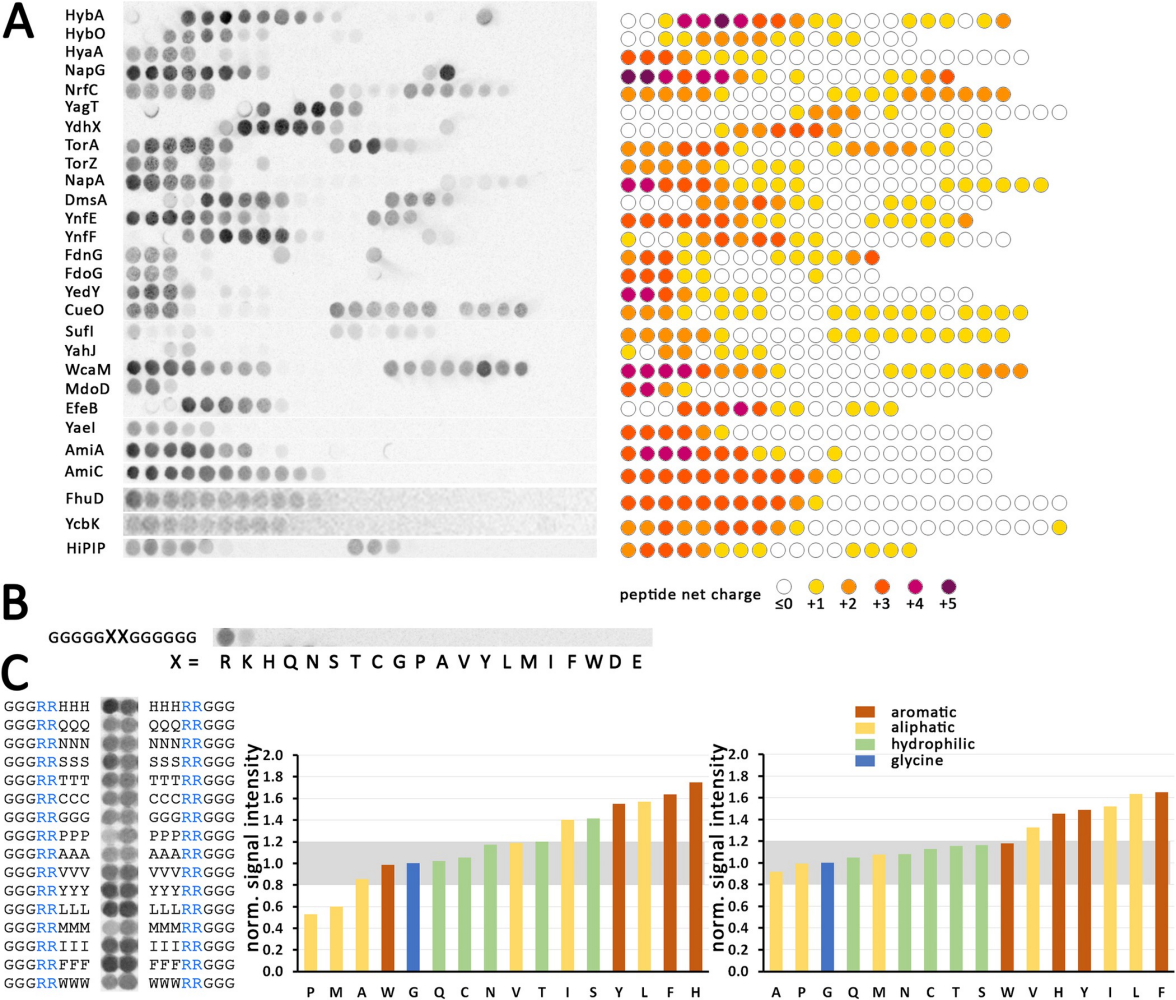
Peptide screen analyses demonstrate selective binding of SlyD to peptides with a positive net charge. A) Screen covering all *E*. *coli* Tat signal peptides and HiPIP, as indicated. See paragraph “peptide spot arrays” in the Methods section for technical details, and supplementary S2 Table for a list of the sequences. Left: Original autoradiogram showing the signals of ^35^S-labeled SlyD. Right: Schematic diagram, showing the peptides with positive net charge in indicated colors. B) Analysis of SlyD-binding to indicated amino acids in a glycine environment. C) Analysis of SlyD-binding to twin-arginines in an indicated uncharged environment. Left: Original data, Right: Quantification of the signals, normalized to the signal obtained with glycine, with bars in the following color code: brown, aromatic; yellow, aliphatic; green polar; blue, glycine (used for normalization).

As several Tat signal peptides have been demonstrated to interact with *E*. *coli* SlyD [13], we carried out a peptide scan, covering the signal peptide sequences of all 27 *E*. *coli* Tat substrates and of the model Tat substrate HiPIP from *Allochromatium vinosum*, to analyze the interaction with SlyD. Peptides of 13 residues length were synthesized in spots on cellulose membranes, from the N-terminal peptide stepwise down to a peptide covering the signal peptide cleavage site (Fig 2A; see S2 Table for list of peptide sequences). ^35^S-labeled SlyD was purified, bound to the peptides, non-bound SlyD was washed off, and binding of SlyD to the spots was analyzed by exposure of the washed membranes to a phosphorimager screen (see Methods for details). All Tat signal peptides contained epitopes that could be bound by SlyD. In all cases, peptides were recognized that contained the twin-arginines in conjunction with hydrophobic residues in the neighborhood. Many signal peptides contained a second binding site at the end of the h-region. Also in these cases, the bound peptides contained a positive net-charge. Notably, lysine-containing peptides were weaker bound than arginine-containing peptides, and binding usually increased with increased positive net charge. As the binding site of SlyD is known to constitute a hydrophobic pocket that accommodates two hydrophobic residues [19], and as charge interactions could not be detected in three out of four crystal structures that were obtained at high salt conditions that can disrupt charge interactions, the mode of the apparent charge interaction was unclear so far. Strikingly, there was not a single peptide without positive net-charge that interacted with SlyD in our experiment.

On one hand, these data indicated that positive charges were required for the interaction, and on the other hand it was likely that this basal binding was intensified by uncharged residues in the environment of the charged positions, as the signal intensity varied between peptides of the same net-charge. To address this aspect, we first examined the binding of SlyD to all individual amino acids, as present in an oligoglycine environment (gggggXXgggggg, Fig 2B). These peptides have a reduced complexity in comparison to the peptides shown in Fig 2A, and therefore can clarify the aspect of the basal charge interaction. The results confirmed that positive charges sufficed for a basal binding of SlyD to the synthesized peptides, with arginines being more efficiently bound than lysines. Such a basal binding that is based on charge interactions is not surprising, as SlyD is an acidic protein. In agreement with the signal peptide screen, hydrophobic residues were not bound in the absence of positively charged residues. As hydrophobic residues are known to be bound by the active site of SlyD [19], the observed charge recognition therefore most likely represented a second mode of interaction, which could add to the binding of hydrophobic residues.

To test this, we analyzed the binding of SlyD to peptides containing a central “RR”, flanked either N-terminally or C-terminally by three uncharged amino acids on one side, and by three glycines on the respective other side. We would like to note that this approach certainly can only give a first clue about which type of residue is preferred over others, and we do not expect that all three flanking residues can or need to enter the active site, as we don’t know at this stage, how exactly which residue in which distance from the positive charges is involved in binding. The signal intensities were quantified using ImageJ [35], and normalized to the value of the arginines in a glycine environment (Fig 2C). As expected for a low-specific binding, charges alone sufficed to obtain a basal interaction. However, aromatic and large aliphatic residues clearly enhanced the electrostatic interactions, and this enhancement likely reflects specific binding to the hydrophobic binding pocket of SlyD. Alanine and proline did not have any enhancing effect, most likely not fitting deep enough into the hydrophobic binding site. Proline, which lacks an amide proton for hydrogen bond formation, also would destabilize secondary structures that are likely to be important at the binding site, which fits our data. Also, methionine did not enhance binding, possibly due to some effect of its thioether, and tryptophane similarly did not enhance binding, likely due to its large dimensions that might not fit into the binding pocket. Considering the fact that hydrophobic residues alone in the Tat signal peptide screen did not suffice to generate an interaction, the results strongly indicated that SlyD binds charged side chains in addition to a range of aliphatic or aromatic side chains residues (F, Y, H, I, L; note that histidine was uncharged under the experimental conditions). This is reminiscent to DnaK, which also binds a subset of hydrophobic residues in neighborhood to positively charged positions and exposes negatively charged surfaces near the hydrophobic binding site [49, 50]. In case of DnaK, best binding is observed when a positively charged residue is in direct vicinity to two hydrophobic residues. Residues K, L, I, W, R, and S are enriched in DnaK binding sites, with positive charges more frequent at the N-terminal border of bound epitopes, and hydrophobic residues enriched in the following positions [49]. This largely resembles the situation we found for SlyD, but DnaK binds W and not F and H, which contrasts SlyD that recognizes F and H, but not W. Notably, DnaK was also found to bind Tat signal peptides [13, 51].

### SlyD binds the signal peptide of a fully folded Tat substrate with high affinity and has a preference for arginines

**Fig 3:**
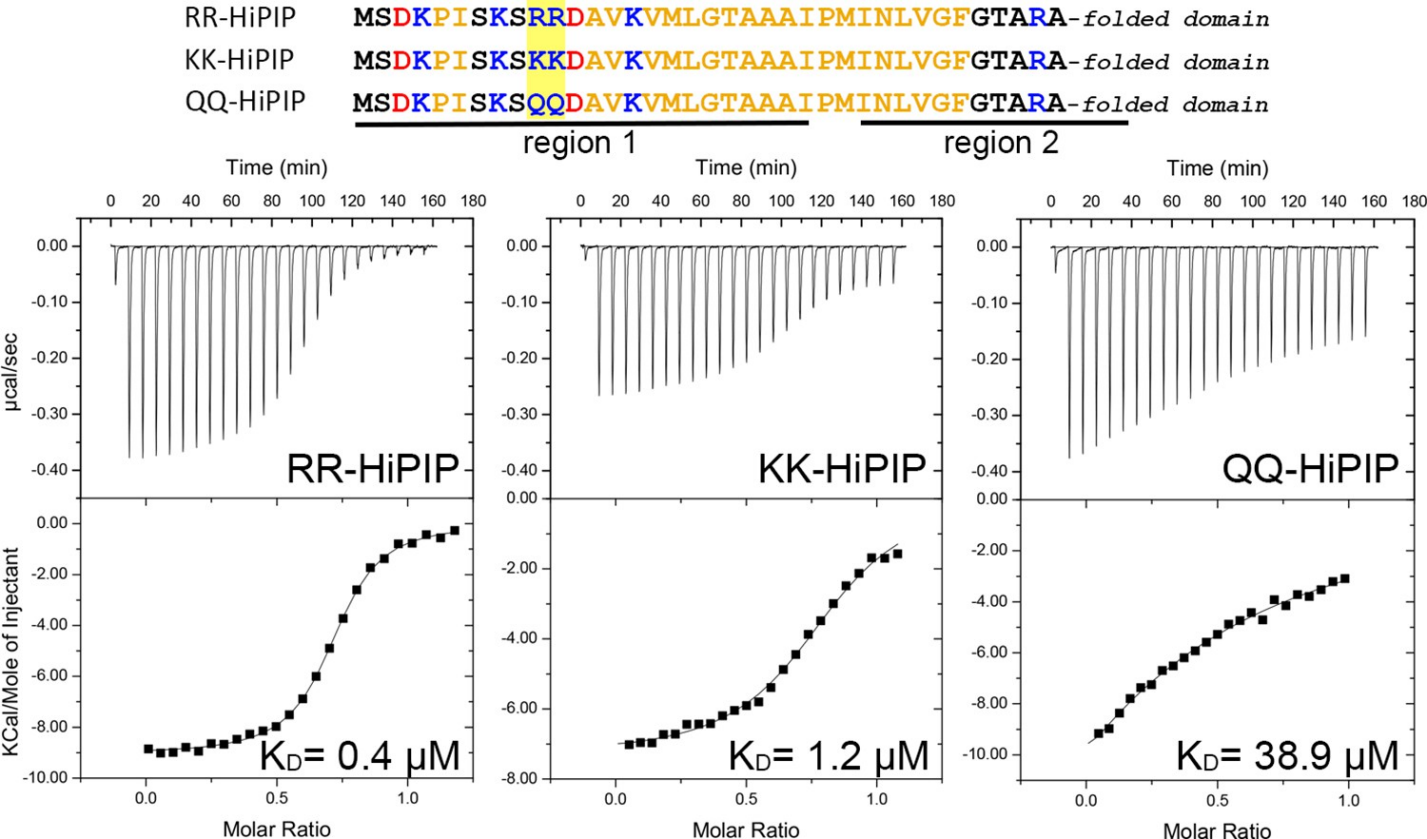
SlyD binds wild type HiPIP precursor (RR-HiPIP) with higher affinity than the HiPIP precursor with an RR>KK or RR>QQ exchange. ITC analysis of SlyD-binding to RR-HiPIP, KK-HiPIP, and QQ-HiPIP. K_D_ values are indicated. See Methods for details. The signal peptides of the analyzed proteins are shown at the top of the figure, with the two potential SlyD-binding regions as identified in the peptide scan (Fig 2) and the twin-arginines underlined, and residues colored according to their properties (blue: positive; red: negative; ocher: hydrophobic; black: all other).

In the first structural study on *E*. *coli* SlyD, the binding of the model Tat substrate HiPIP has been used as one of several proteins and peptides that are bound by SlyD [3]. The only reported K_D_ for a Tat signal peptide/SlyD interaction, which was ~0.1 μM, has been obtained by tryptophane fluorescence changes in response to peptide titration, using *T*. *thermophilus* SlyD in combination with a synthetic *E*. *coli* CueO Tat signal peptide [19]. To characterize the binding of Tat substrates to *E*. *coli* SlyD in more detail, we carried out isothermal titration calorimetry (ITC), using HiPIP as substrate, which contains the signal peptide that had been used for the NMR titration experiments reported earlier [3]. As the peptide arrays had indicated a preference for arginines, which are conserved in the RR-motif of Tat signal peptides, we included a mutated RR>KK variant of HiPIP in our ITC analyses. These experiments showed that the K_D_ of the interaction with wild type HiPIP was at ~0.35 ± 0.02 μM, whereas the K_D_ of the interaction with the KK-HiPIP was at 1.18 ± 0.09 μM, confirming that the twin-arginines indeed enhanced the interaction in comparison to twin-lysines, but KK-HiPIP was still bound (Fig 3). The stoichiometry was N ~ 0.7–0.8 in both cases, leaving it unclear whether one or two binding sites could be bound by SlyD. The peptide scan had shown for HiPIP that peptides were recognized that covered the RR motif and beside this, also peptides were recognized that included the positive charge in the c-region. These two potential binding regions are indicated in the sequences shown in Fig 3. To clarify whether or not the second binding site is used, we also analyzed a QQ-HiPIP variant, in which the twin-arginines of the signal peptide were exchanged by glutamines, thereby substituting the charged residues by polar but uncharged side chains (Fig 3). Notably, this exchange reduced the affinity by at least 100-fold, showing a K_D_ of 38.7 ± 2 μM. This low-affinity binding may well be mediated by single lysine residues that are present in close proximity to the mutated twin-arginine motif, and thus may become an accessible 2^nd^ choice binding site when the twin-arginines are mutated. Clearly, the twin-arginine region is therefore the only relevant binding site, and we can conclude that HiPIP interacts with SlyD in a 1:1 ratio, which also fits to the simple sigmoidal curves seen in ITC. The reason for the aberrant N-values, which imply potential binding of more than one SlyD to HiPIP, is likely due to an overestimation of the SlyD concentration, as the calculated extinction coefficient Ɛ_280nm_ = 6.335 mM^-1^ cm^-1^ has been taken for concentration determinations, and this theoretical value is likely not exact. Our SlyD was >99% pure as judged by Coomassie-stained SDS-PAGE gels, with only one additional band detectable that could be assigned as a dimer by Western blotting using SlyD-specific antibodies (S3 Fig). Therefore, we are convinced that the assumed theoretical extinction coefficient resulted in some over-estimation of the SlyD concentration, which was even variable, as extinction coefficients may have been influenced by mutations or bound nickel in some cases.

The interaction with RR- and KK-HiPIP variants was largely enthalpy-driven, with ΔH ~ -9,060 cal mol^-1^ and -TΔS ~ 303 cal mol^-1^ deg^-1^ found for RR-HiPIP, and ΔH ~ -7,700 cal mol^-1^ and -TΔS ~ 891 cal mol^-1^ deg^-1^ in case of KK-HiPIP, in agreement with the preference for arginines over lysines, as observed in the peptide screens.

### Some functionality defects caused by IF domain mutations correlate with reduced substrate affinity

**Fig 4:**
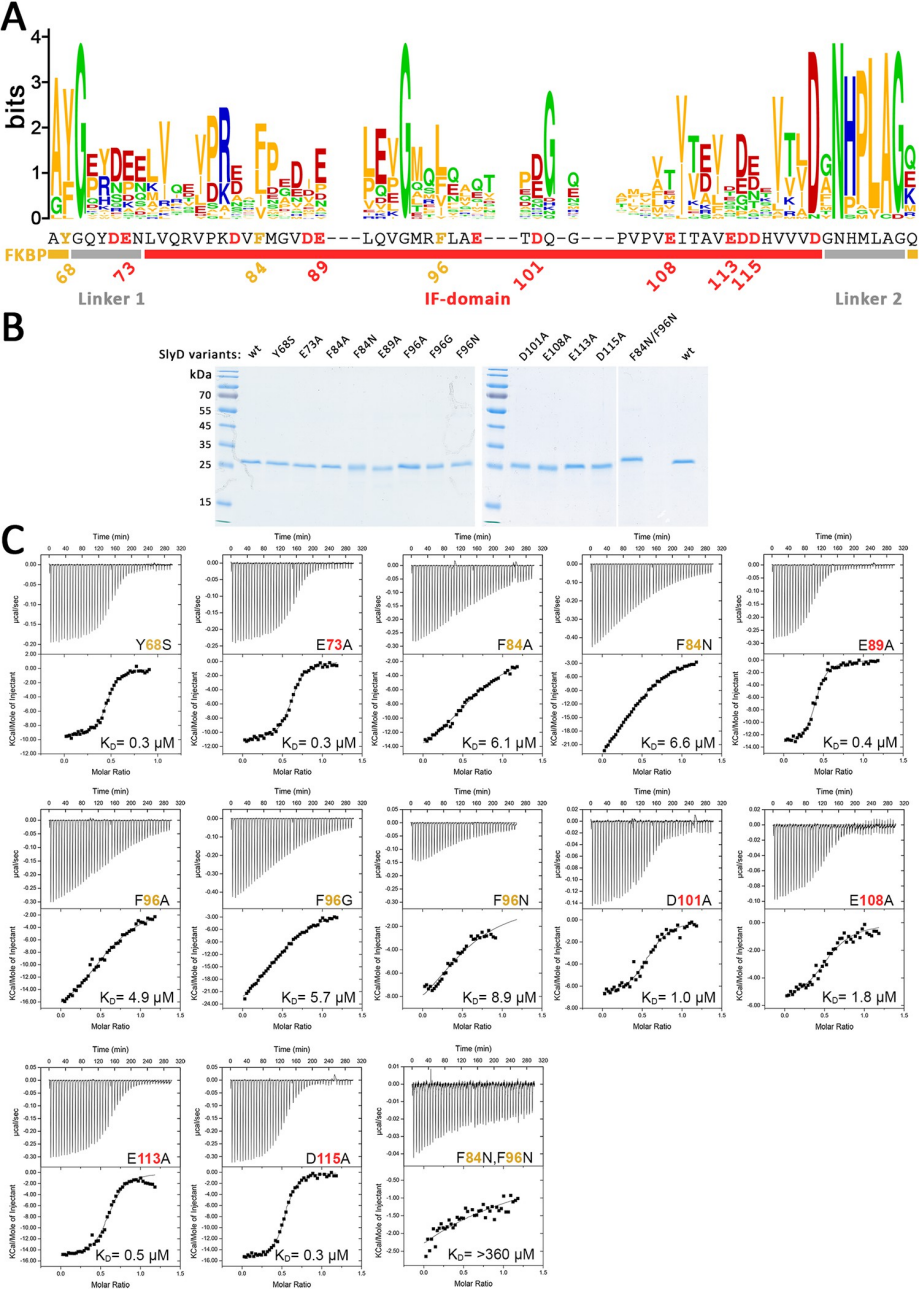
Mutation of charged residues have less effect on substrate affinity than mutations of the active site phenylalanines. ITC analyses of mutated SlyD variants A) WebLogo [44] analysis of an alignment of SlyD sequences from a wide range or organisms (see S1 Fig and S1 Table). The alignment was done using Clustal-Omega [43]. B) SDS PAGE analysis of purified SlyD variants (Coomassie stain); C) ITC titration of indicated SlyD variants. See S3 Table for complete data set.

While the hydrophobic pocket in the SlyD chaperone domain captures two hydrophobic residues of the target sequence to a confined position [19], the remainder of bound peptides can access a large area of the domain surface. To address potential effects of removals of negative charges, we carried out ITC titrations with RR-HiPIP that analyzed the SlyD variants with exchanges in representative positions, distributed all over the surface, including E73A, E89A, D101A, E108A, E113A, and D115A. As positive controls for mutations with effects on IF domain function, we also included F84A, F84N, F96A, F96G, and F96N, as well as a combination of F84N/F96N exchanges in our analyses (positions indicated in Fig 4A). So far, such mutations directly in the hydrophobic pocket of the chaperone have not been analyzed, and the role of these residues in binding has been inferred from structural data, but it was expected that the exchange of these key hydrophobic residues should have strong effects of substrate-binding. The F84A and F96A exchanges remove the phenyl group and leave just a methyl group, the F96G removes the whole side chain and further increases flexibility, and the F84N and F96N exchanges substitute the aromatic by an uncharged and not too small polar side chain that can be accommodated. As negative control, i.e. as mutation that is not expected to have an effect on IF domain function, we included the Y68S mutation in our analysis, a position important for substrate-binding to the prolyl isomerase active site [28] whose mutation should not affect binding if only the chaperone active site is responsible for Tat signal peptide binding. To obtain large amounts of all SlyD variants, we used a stronger expression system, obtained C-terminally His-tagged SlyD in inclusion bodies, followed by refolding and purification by affinity chromatography (see Methods). All SlyD variants were stable in solution and purified to apparent homogeneity, as judged by SDS-PAGE/Coomassie staining (Fig 4B).

The ITC results are shown in Fig 4C. The combined F84N/F96N exchanges resulted in the strongest binding defect, not permitting any reliable determination of binding parameters anymore, confirming that the hydrophobic binding site plays a central role in substrate binding. Although all SlyD variants were stable in solution, the completely abolished interaction with the HiPIP substrate in case of the F84N/F96N variant may well be due to significant effects on flexibility and stability of the IF domain. With respect to the single mutations in the hydrophobic active site, the effect increased in the order F84A < F84N, and F96A < F96G / F96N, showing that hydrophobicity at these positions is indeed important. As the affinity of these single-exchange variants is still in the 4–10 μM range, the binding site must still be formed, but the decrease in affinity suggests that hydrophobic interactions with the substrate and/or the conformational flexibility of the binding site are affected. The Y68S variant did not significantly affect the K_D_. This, together with the abolishment of SlyD-binding by the F84N/F96N double exchange, demonstrated that only the IF domain is relevant for binding of the HiPIP signal peptide, and not the FKBP domain, which fully agrees with the evidence that has been obtained by NMR and fluorescence techniques [3, 19].

**Fig 5:**
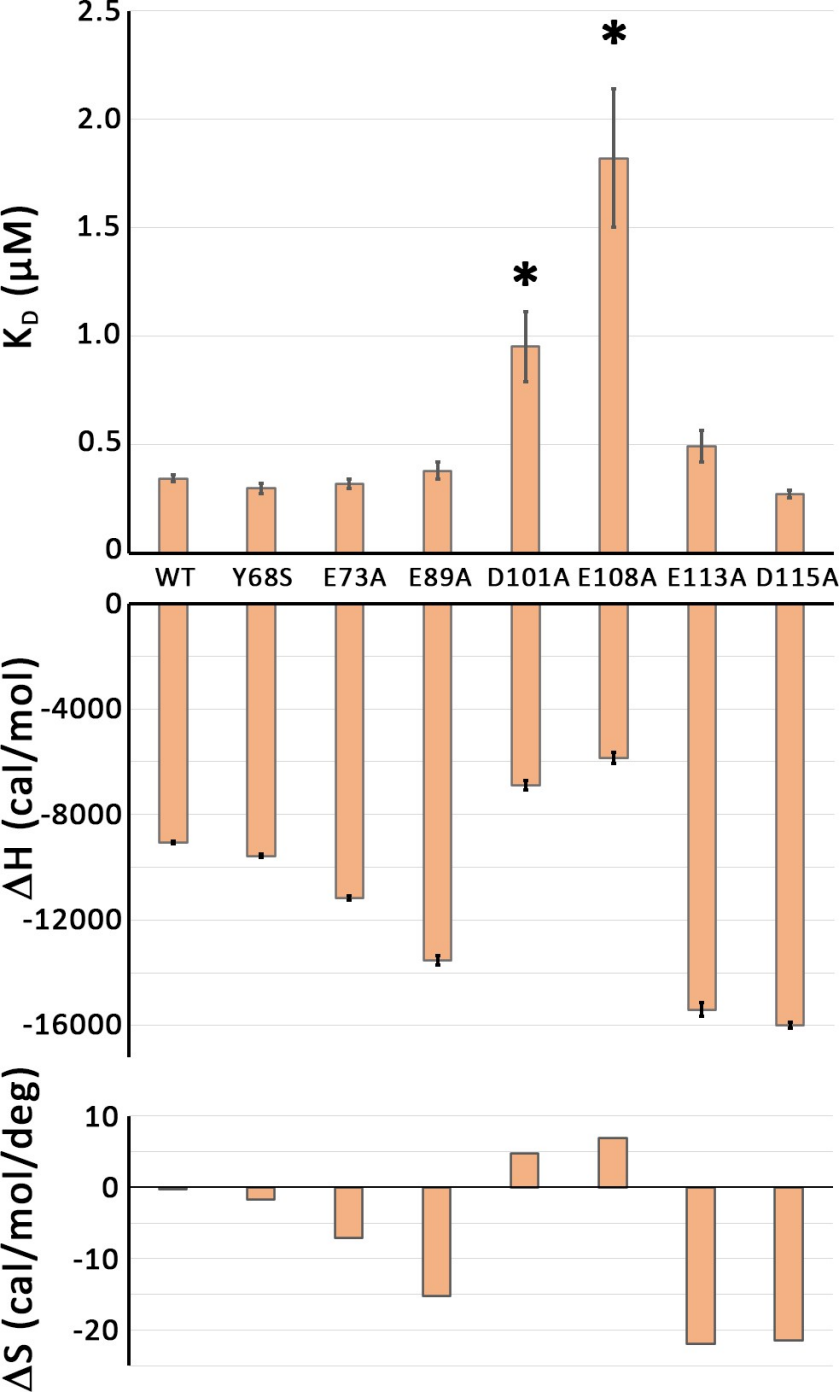
Two of the SlyD variants with exchanged negative charges are significantly affected in HiPIP signal peptide-binding. Plots of K_D_, ΔH, and ΔS for the SlyD variants with indicated exchanged negatively charged residues. The data from the wild type and the Y68S variant were included as negative controls. See text for details.

The ITC analyses of the SlyD variants with mutated charged positions are shown in more detail in Fig 5. Notably, two SlyD variants with exchanges of charged residues showed a weaker binding (K_D_ (D101A) = 0.95 μM; K_D_ (E108A) = 1.82 μM), indicating that these charged residues contribute to binding. The effect was mainly due to enthalpy differences (see ΔH and ΔS plots in Fig 5). The other exchanges of charged residues did not reduce the affinity, suggesting that the model substrate HiPIP interacts with the chaperone surface near D101 and E108.

### The negatively charged surface of IF domains is highly conserved

**Fig 6:**
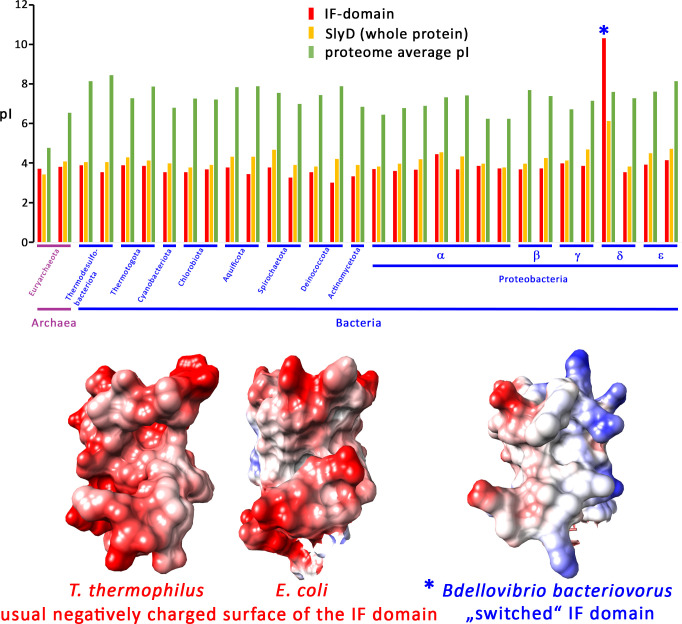
A highly negative IF domain surface is conserved among SlyD orthologs in archaea and bacteria. A) pI values of SlyD IF domains from species of indicated phyla or classes, in comparison to the overall pI of SlyD and the average pI of the whole proteome of the respective organism (see Methods for details). B) Electrostatic surface charge characteristics of SlyD IF domains from *T*. *thermophilus* and *E*. *coli*, as examples of typical acidic SlyD IF domains, and from *B*. *bacteriovorus*, the only to us known example of a “switched” alkaline SlyD IF domain. Only the IF domains are shown for clarity reasons. See text for details.

As our data showed that the recognition of positively charged residues is important for the SlyD interaction, although there is not much conservation of individual negatively charged residues on the IF domain surface, it became a likely scenario that rather a more general negative electrostatic potential at the surface might be important, and variations of the surface charges might reflect adaptations to the set of natural substrates in specific organisms. To analyze this aspect further, we examined the pI of IF domains from SlyD orthologs of many diverse phyla (Fig 6). Notably, IF domains are usually highly acidic, with a pI of around 4, which is in average slightly more acidic than the overall pI of SlyD, and these “*acidic SlyDs*” have a much more acidic pI than the average pI of the respective whole proteome (Fig 6). We found one revealing exception, which is SlyD from *Bdellovibrio bacteriovorus*, a parasitic δ-proteobacterium with a unique life style, as it reproduces inside other bacteria [52]. Specifically, its IF domain was switched from highly acidic to highly alkaline by more than 6 orders of magnitude, while the rest of the protein was not. This case strongly suggests that the bound targets most likely have changed from cationic peptides to anionic peptides in this organism, and the extreme change underlines the importance of electrostatic interactions in the overall function of the SlyD IF domain. Fig 6 compares in its lower part the electrostatic surface characteristics of IF domains from two “*acidic SlyDs*” (exemplified by the best characterized *E*. *coli* and *T*. *thermophilus* SlyDs) directly with the “*alkaline SlyD*” from *B*. *bacteriovorus*. The comparison reveals that an IF-domain-specific switch of the electrostatic surface characteristics has taken place in evolution in *Bdellovibrio*, which further supports the proposed role of the electrostatic surface properties of the IF domain in substrate binding.

## Discussion

SlyD is a general chaperone that is known to play an important role in the biogenesis of nickel-containing redox enzymes, being actively involved in nickel delivery to the metal centers of these enzymes [9]. However, we found that many SlyD homologs miss the C-terminal nickel-binding domain, including archaeal species and members of the phyla Thermodesulfobacteriota, Thermotogota, Cyanobacteriota, Chlorobiota, and some branches of the α- and δ-proteobacteria (S1 Fig), indicating that nickel incorporation into enzymes cannot be the only function of SlyD orthologs. SlyD also binds to the nickel permease Niu and thereby regulates nickel uptake [17]. Moreover, it is now known that SlyD is also involved in nickel-unrelated pathways: SlyD has been recently found to be involved in efficient iron-sulfur cluster assembly for enzymes of the TCA cycle [53], and SlyD-binding to the signal peptide of the copper-resistance protein CueO is important for the transport of this Cu^I^-oxidase to the periplasm of *Campylobacter jejuni* [15]. However, in *E*. *coli*, CueO transport and copper resistance does not depend on SlyD ([13], and S2 Fig), indicating that this Tat-related function in *Campylobacter jejuni* is not conserved.

**Fig 7:**
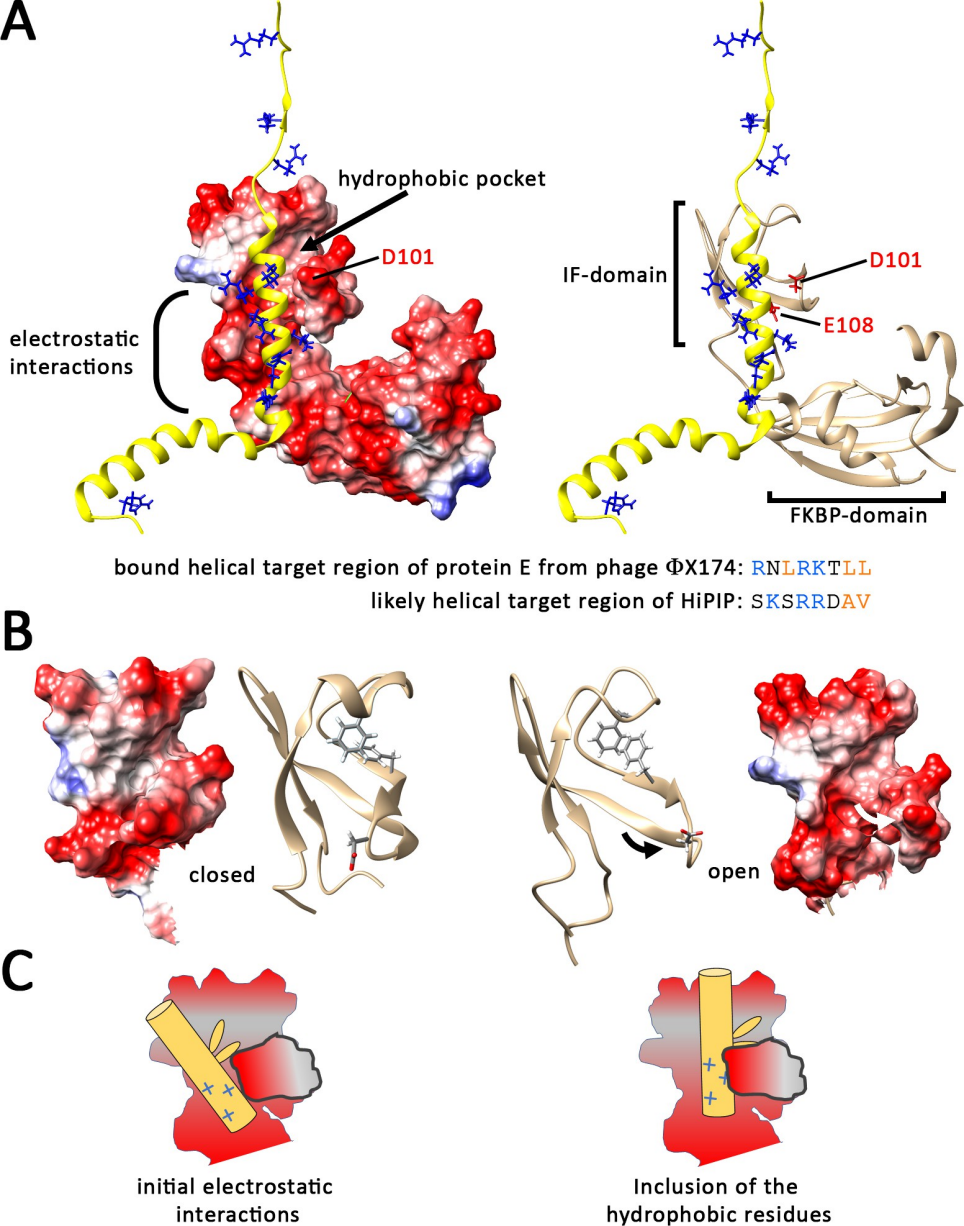
Model for the binding of helical signal peptides by SlyD. A) The SlyD-bound protein E of phage ΦX174 (yellow) aligns its α-helix in a way that uses the hydrophobic pocket and adjacent negatively charged surface for the interaction (PDB 8G02). Positively charged residues of protein E are shown and colored in blue. The electrostatic potential is indicated in the left image (red: negative, blue: positive), whereas the right image facilitates the recognition of the secondary and tertiary structures that are involved. The IF and FKBP domains are labeled and their relative orientations are different from the structure shown in Fig 1, which is due to the flexible linker region that connects both domains. Note that the bound region of protein E resembles the twin-arginine motif region of HiPIP that is bound by SlyD. B) Comparison of SlyD IF domain structures as found when no substrate is bound (closed; PDB 2K8I) or when the helical substrate protein E is bound (open; substrate hidden; PDB 8G02). A conformational change of two beta-strands that opens access to the hydrophobic pocket from a vertically aligned α-helix is induced by binding of protein E to SlyD. The positions of the hydrophobic pocket residues F84 and F96 are shown, as well as the position of the D101 residue that is at the tip beta sheet that opens upon substrate interaction. Note that the orientation of F96 changes from the closed to the open state. C) Schematic binding modes for helical peptides to the IF domain. An initial electrostatic interaction induces a conformational change that permits binding of the hydrophobic residues by SlyD.

Our data presented now demonstrate that the positive charge of bound peptides is of key importance for SlyD binding. Arginines are preferred over lysines, but both charged residues can serve the purpose of supporting binding if the bound region has a positive net charge (Figs 2 and 3). All peptides that have been found to bind SlyD with high affinity were positively charged [19], and also the recently published first structure of a SlyD with a bound protein substrate, the protein E of phage ΦX174, confirmed this aspect, as the bound protein E is highly positively charged ([8] and Fig 7A). There are multiple ways by which peptides that can form either β-strands [19] or α-helices [8] can be bound to SlyD, which is an impressive characteristic of the SlyD chaperone. SlyD can thus function in a multitude of biological pathways, and the IF domain can contribute to these functions as long a hydrophobic region with neighboring positive charges is provided. While the prolyl isomerase activity is physiologically important for protein folding and can be enhanced by the IF domain [25], SlyD apparently can bind peptides such as the small protein E and prevent their degradation [7], which may be the major function for SlyD- and DnaK-binding to Tat signal peptides [13].

Binding of Tat signal peptides to *E*. *coli* SlyD has been studied by NMR, which indicated that the IF domain is responsible for the interaction [3]. The affinity to a synthetic Tat signal peptide has been determined by ITC also with *T*. *thermophilus* SlyD, and a K_D_ of ca. 0.11 μM was found, which is similar to our results [19]. These authors used a higher salt concentration, whereas we used a rather low salt concentration, which may facilitate the detection of ionic interactions. It must be noted that ITC does not precisely mimic *in vivo* cytoplasmic conditions, such as the reducing environment or the macromolecular crowding with high protein or nucleic acid concentrations. Notably, the SlyD interaction with the peptide array resisted high salt washes, indicating that the ionic interactions are quite strong.

Since structural details are not determined, it can only be hypothesized how exactly the interaction takes place. As Tat signal peptides are unfolded in solution but can form α-helices and are postulated to form α-helices upon contact to membranes or other proteins [54, 55], it is likely that their binding to SlyD resembles the interaction of protein E with phage ΦX174 (Fig 7). Notably, the bound helix of protein E influenced the orientation of secondary structures in the IF domain, generating a clamp in which negatively charged IF domain surface regions play a role (Fig 7A). Two beta sheet strands with D101 at their turn form a kind of thumb that is contacting the positive charges, and at the same time these beta sheets contribute to the hydrophobic pocket. Comparison with the SlyD structure without bound substrate reveals a movement of these beta strands in response to binding of the helical substrate, which opens the binding site and facilitates access to the hydrophobic pocket, in which residues such as F96 can alter their orientation (Fig 7B). Such a structural transition is in well agreement with the multiple changes that have been seen in response to HiPIP signal peptide binding to SlyD by NMR [3]. The electrostatic interactions thus likely initiate the “induced fit” that results in tight binding of the substrate by SlyD, as schematically outlined in Fig 7C. This mechanism would explain our observation that charge interactions are essential for binding (Fig 2), and that a larger negatively charged surface rather than a single specific side chain is important for these interactions (Figs 5 and 6). An initial electrostatic interaction that is followed by a hydrophobic effect-driven tighter binding appears to be a rather common theme in substrate-binding by chaperones [56, 57]. Future structural studies will be required to address this hypothesis for the SlyD mechanism.

## Supporting information

S1 Fig Phylogenetic tree based on the sequences of bacterial and archaeal SlyD from diverse phyla. SlyD orthologs that lack a C-terminal histidine-rich metal-binding domain are indicated.
(TIF)

S2 Fig The absence of SlyD does not affect copper resistance of *E*. *coli*. Cultures were grown aerobically overnight in LB medium, and 1 ml of cells adjusted to OD600 = 1 were washed three times in 1.5 mL saline (0.9% [w/v] NaCl). The final pellet was resuspended in 1 mL saline solution and 5 μL droplets of serial dilutions diluted 1:10 up to 10–6 with saline were placed on M9 media agar plates supplemented with 2 μM FeCl3 and indicated concentrations or CuSO4, and grown aerobically overnight. Strains used: wt, MC4100; Δ*slyD*, MC4100 Δ*slyD*; Δ*cueO*, MC4100 Δ*cueO*.
(TIF)

S3 Fig Folded state and purity of SlyD used for ITC analyses. A) Circular dichroism spectrum of SlyD, showing the minimum at 215 nm and shoulder at 228 nm, indicative for the apo-form of SlyD (1) (conc.: 4.4 μM, path length 0.1 cm, sample temperature 20°C, data interval 1 nm, scanning speed 50 nm/min, 1 nm bandwidth, 10 scans averaging; measured with JASCO J-815 spectropolarimeter) B) SDS-PAGE/Western blot analysis and Coomassie-stained SDS-PAGE gel of indicated amounts of purified histidine-tagged SlyD, showing the high purity of the sample and traces of dimeric associations. The blot was developed with SlyD-specific antibodies (gift of Cordelia Schiene-Fischer, University of Halle). M, marker proteins. Molecular masses of marker proteins are indicated on the left.
(TIF)

S4 Fig A Strep-tagged SlyD has the same affinity for HiPIP as His-tagged SlyD, indicating that the tag fused to the C-terminal domain has no effect on substrate binding by the IF domain. ITC-measurement with 29 μM C-terminally Strep-tagged SlyD titrated with 166 μM RR-HiPIP (18 injections of 10 μl, preceded by a 2 μl injection).
(TIF)

S1 Table SlyD proteins used for the phylogenetic tree in S1 Fig, the Clustal-Omega/WebLogo analysis shown in Fig 4A, and the pI analyses shown in Fig 6A. (PDF)

S2 Table Sequences of the Tat signal peptide screen shown in Fig 2. Each line gives the sequence of an individual peptide that has been synthesized on a spot. Names of the respective Tat substrates are indicated above the corresponding peptide sequences.
(PDF)

S3 Table N-values, affinities and enthalpies of SlyD interactions with HiPIP precursor (RR-HiPIP), as derived from ITC. The asterisk (*) marks output data that reflect no specific interaction.
(PDF)

S1 Raw data (XLSX)

S1 Raw images (PDF)
