# Supplementary figures and images for: Positive charges promote the recognition of proteins by the chaperone SlyD from Escherichia coli
Source: PLoS One. 2024 Jun 25;19(6):e0305823. doi: 10.1371/journal.pone.0305823 (PMC11198818; doi:10.1371/journal.pone.0305823)

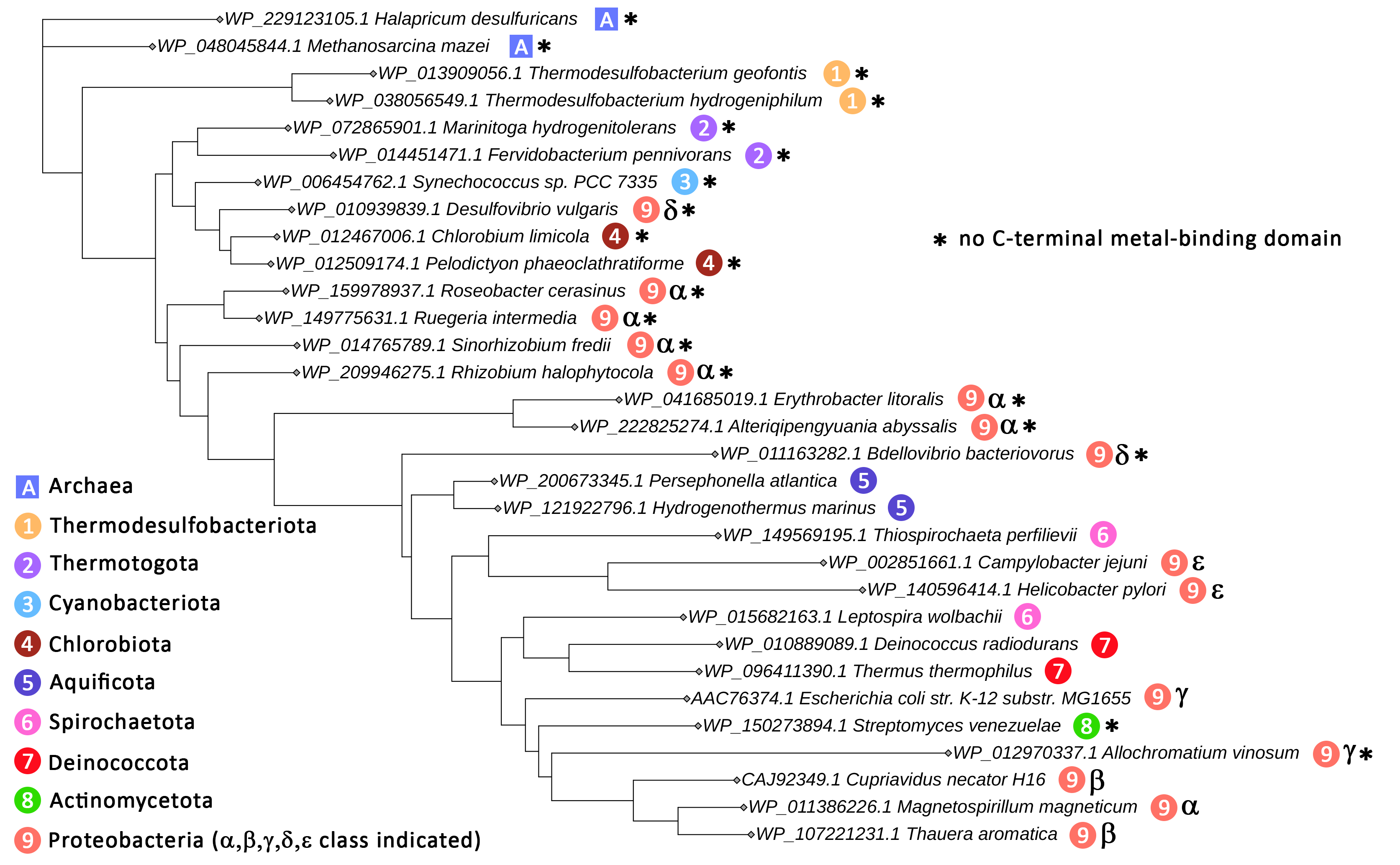

Supplement: S1 Fig — SlyD orthologs that lack a C-terminal histidine-rich metal-binding domain are indicated. (TIF) [file pone.0305823.s001.tif]

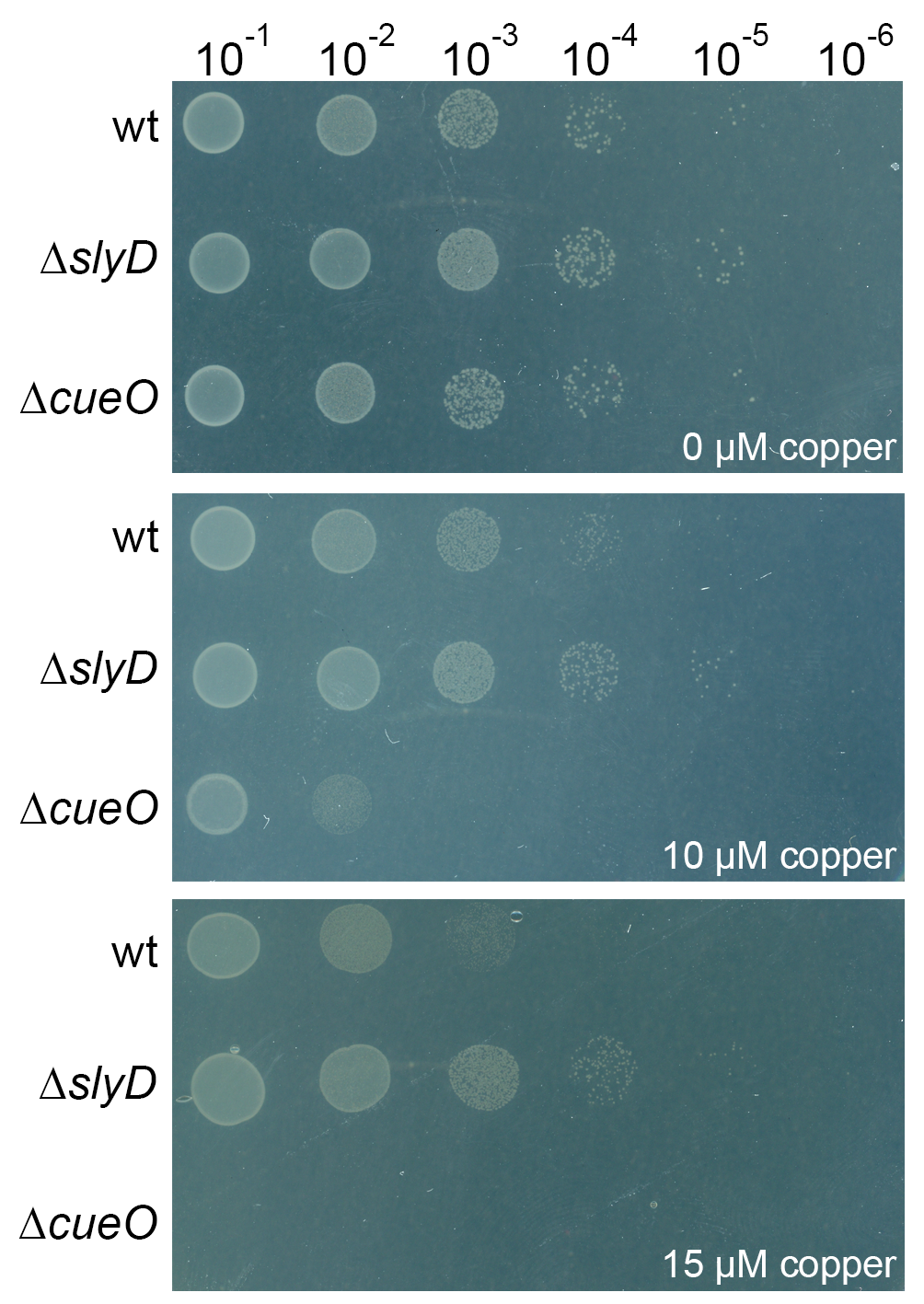

Supplement: S2 Fig — Cultures were grown aerobically overnight in LB medium, and 1 ml of cells adjusted to OD600 = 1 were washed three times in 1.5 mL saline (0.9% [w/v] NaCl). The final pellet was resuspended in 1 mL saline solution and 5 μL droplets of serial dilutions diluted 1:10 up to 10–6 with saline were placed on M9 media agar plates supplemented with 2 μM FeCl3 and indicated concentrations or CuSO4, and grown aerobically overnight. Strains used: wt, MC4100; ΔslyD, MC4100 ΔslyD; ΔcueO, MC4100 ΔcueO. (TIF) [file pone.0305823.s002.tif]

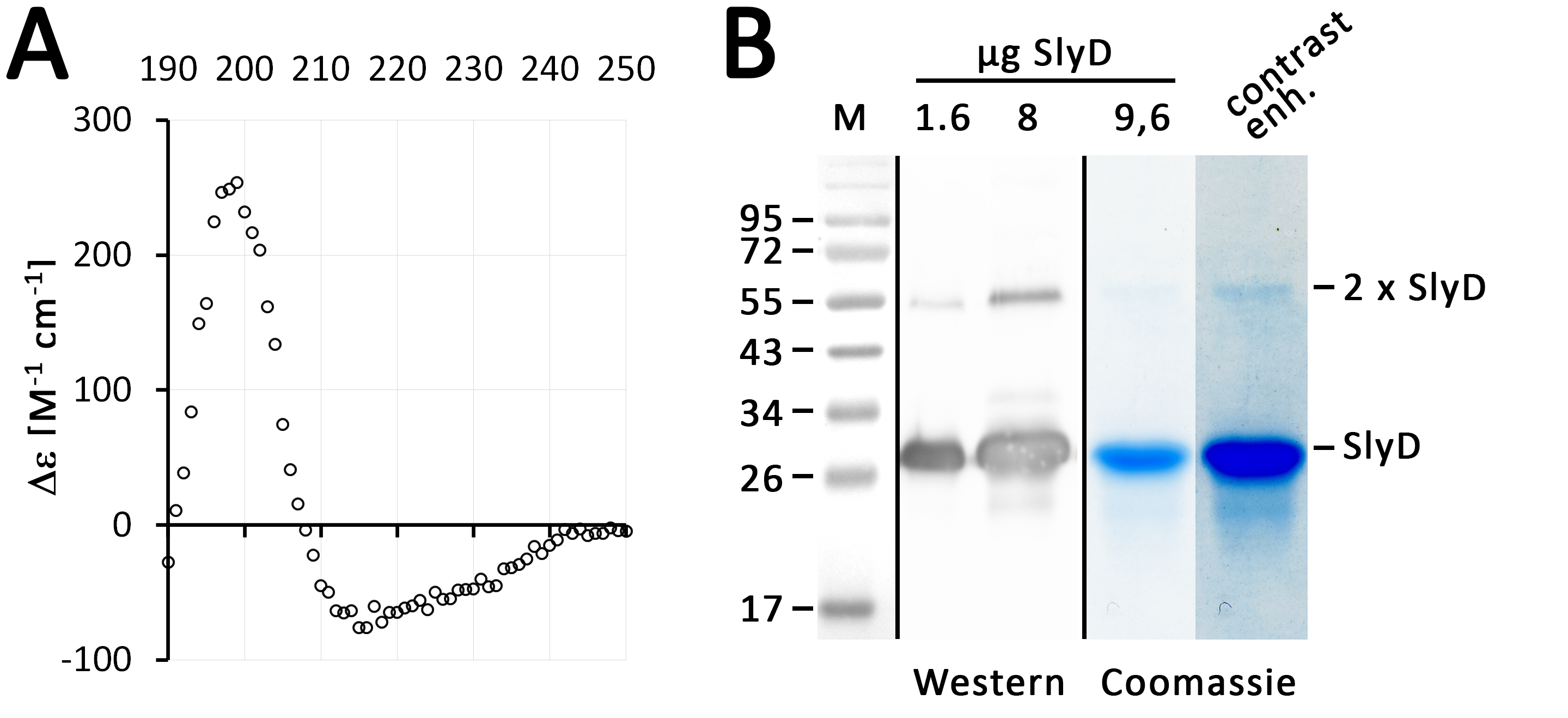

Supplement: S3 Fig — A) Circular dichroism spectrum of SlyD, showing the minimum at 215 nm and shoulder at 228 nm, indicative for the apo-form of SlyD (1) (conc.: 4.4 μM, path length 0.1 cm, sample temperature 20°C, data interval 1 nm, scanning speed 50 nm/min, 1 nm bandwidth, 10 scans averaging; measured with JASCO J-815 spectropolarimeter) B) SDS-PAGE/Western blot analysis and Coomassie-stained SDS-PAGE gel of indicated amounts of purified histidine-tagged SlyD, showing the high purity of the sample and traces of dimeric associations. The blot was developed with SlyD-specific antibodies (gift of Cordelia Schiene-Fischer, University of Halle). M, marker proteins. Molecular masses of marker proteins are indicated on the left. (TIF) [file pone.0305823.s003.tif]

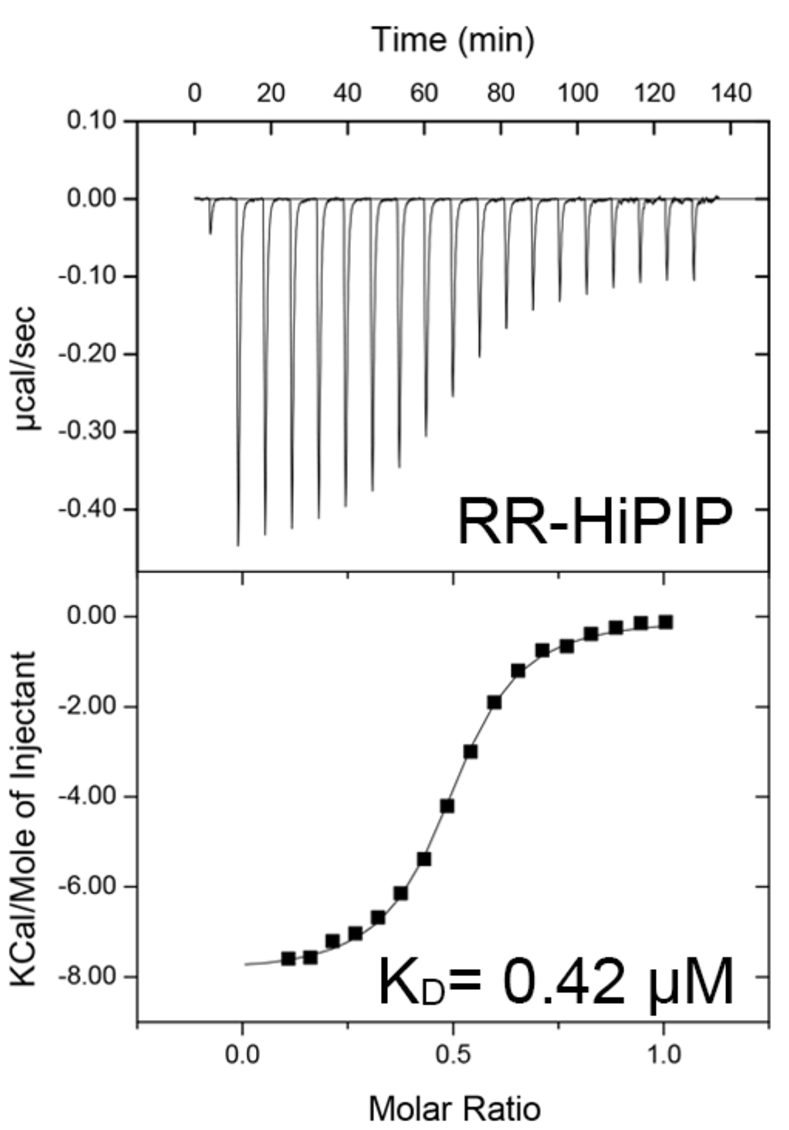

Supplement: S4 Fig — ITC-measurement with 29 μM C-terminally Strep-tagged SlyD titrated with 166 μM RR-HiPIP (18 injections of 10 μl, preceded by a 2 μl injection). (TIF) [file pone.0305823.s004.tif]
