# Supplementary material for: Positive charges promote the recognition of proteins by the chaperone SlyD from Escherichia coli
Source: PLoS One. 2024 Jun 25;19(6):e0305823. doi: 10.1371/journal.pone.0305823 (PMC11198818; doi:10.1371/journal.pone.0305823)
Supplement: S1 Table — (PDF) [file pone.0305823.s005.pdf]

S1 Table. SlyD proteins used for the phylogenetic tree in S1 Fig, the Clustal-Omega/WebLogo analysis shown in Fig 4A, and the pI analyses shown in Fig 6A.

Organism name	Annotation	GenBank ID	Assembly ID
Allochromatium vinosum	peptidyl-prolyl cis-trans isomerase	WP_012970337.1	GCA_000025485.1
Alteriqipengyuania abyssalis	FKBP-type peptidyl-prolyl cis-trans isomerase	WP_222825274.1	GCA_019857185.1
Bdellovibrio bacteriovorus	FKBP-type peptidyl-prolyl cis-trans isomerase	WP_011163282.1	GCA_000196175.1
Campylobacter jejuni	peptidylprolyl isomerase	WP_002851661.1	GCA_000009085.1
Chlorobium limicola	peptidylprolyl isomerase	WP_012467006.1	GCA_000020465.1
Cupriavidus necator	FKBP-type peptidyl-prolyl cis-trans isomerase	CAJ92349.1	GCA_000009285.2
Deinococcus radiodurans	peptidylprolyl isomerase	WP_010889089.1	GCA_000008565.1
Desulfovibrio vulgaris	peptidylprolyl isomerase	WP_010939839.1	GCA_000195755.1
Erythrobacter litoralis	peptidylprolyl isomerase	WP_041685019.1	GCA_000013005.1
Escherichia coli	FKBP-type peptidyl-prolyl cis-trans isomerase SlyD	AAC76374.1	GCA_000005845.2
Fervidobacterium pennivorans	peptidylprolyl isomerase	WP_014451471.1	GCA_000235405.3
Halapricum desulfuricans	peptidylprolyl isomerase	WP_229123105.1	GCA_017094465.1
Helicobacter pylori	peptidylprolyl isomerase	WP_140596414.1	GCA_006439645.1
Hydrogenothermus marinus	peptidylprolyl isomerase	WP_121922796.1	GCA_003688665.1
Leptospira wolbachii	peptidylprolyl isomerase	WP_015682163.1	GCA_000332515.2
Magnetospirillum magneticum	peptidylprolyl isomerase	WP_011386226.1	GCA_000009985.1
Marinitoga hydrogenitolerans	peptidylprolyl isomerase	WP_072865901.1	GCA_900129175.1
Methanosarcina mazei	peptidylprolyl isomerase	WP_048045844.1	GCA_000007065.1
Pelodictyon phaeoclathratiforme	peptidylprolyl isomerase	WP_012509174.1	GCA_000020645.1
Persephonella atlantica	peptidylprolyl isomerase	WP_200673345.1	GCA_016617615.1
Rhizobium halophytocola	peptidylprolyl isomerase	WP_209946275.1	GCA_017873095.1
Roseobacter cerasinus	peptidylprolyl isomerase	WP_159978937.1	GCA_009811755.1

Organism name	Annotation	GenBank ID	Assembly ID
Ruegeria intermedia	peptidylprolyl isomerase	WP_149775631.1	GCA_900129345.1
Sinorhizobium fredii	peptidylprolyl isomerase	WP_014765789.1	GCA_000265205.2
Streptomyces venezuelae	peptidylprolyl isomerase	WP_150273894.1	GCA_008642375.1
Synechococcus sp.	peptidylprolyl isomerase	WP_006454762.1	GCA_000155595.1
Thauera aromatica	peptidylprolyl isomerase	WP_107221231.1	GCA_003030465.1
Thermodesulfobacterium geofontis	peptidylprolyl isomerase	WP_013909056.1	GCA_000215975.1
Thermodesulfobacterium hydrogeniphilum	peptidylprolyl isomerase	WP_038056549.1	GCF_000746255.1
Thermus thermophilus	peptidylprolyl isomerase	WP_096411390.1	GCA_019974155.1
Thiospirochaeta perfilievii	FKBP-type peptidyl-prolyl cis-trans isomerase	WP_149569195.1	GCA_008329945.1
