# Supplementary material for: Positive charges promote the recognition of proteins by the chaperone SlyD from Escherichia coli
Source: PLoS One. 2024 Jun 25;19(6):e0305823. doi: 10.1371/journal.pone.0305823 (PMC11198818; doi:10.1371/journal.pone.0305823)
Supplement: S2 Table — Each line gives the sequence of an individual peptide that has been synthesized on a spot. Names of the respective Tat substrates are indicated above the corresponding peptide sequences. (PDF) [file pone.0305823.s006.pdf]

S2 Table. Sequences of the Tat signal peptide screen shown in Fig 2. Each line gives the sequence of an individual peptide that has been synthesized on a spot. Names of the respective Tat substrates are indicated above the corresponding peptide sequences.

HyaA<br>M N N E E T F Y Q A M R R<br>N E E T F Y Q A M R R Q G<br>E T F Y Q A M R R Q G V T<br>F Y Q A M R R Q G V T R R<br>Q A M R R Q G V T R R S F<br>M R R Q G V T R R S F L K<br>R Q G V T R R S F L K Y C<br>G V T R R S F L K Y C S L<br>T R R S F L K Y C S L A A<br>R S F L K Y C S L A A T S<br>F L K Y C S L A A T S L G<br>K Y C S L A A T S L G L G<br>C S L A A T S L G L G A G<br>L A A T S L G L G A G M A<br>A T S L G L G A G M A P K<br>S L G L G A G M A P K I A<br>G L G A G M A P K I A W A<br>G A G M A P K I A W A L E<br>G M A P K I A W A L E N K<br>A P K I A W A L E N K P R	HybO<br>M T G D N T L I H S H G I<br>G D N T L I H S H G I N R<br>N T L I H S H G I N R R D<br>L I H S H G I N R R D F M<br>H S H G I N R R D F M K L<br>H G I N R R D F M K L C A<br>I N R R D F M K L C A A L<br>R R D F M K L C A A L A A<br>D F M K L C A A L A A T M<br>M K L C A A L A A T M G L<br>L C A A L A A T M G L S S<br>A A L A A T M G L S S K A<br>L A A T M G L S S K A A A<br>A T M G L S S K A A A E M<br>M G L S S K A A A E M A E<br>L S S K A A A E M A E S V
HybA<br>M N R R N F I K A A S C G<br>N R R N F I K A A S C G A<br>R R N F I K A A S C G A L<br>R N F I K A A S C G A L L<br>N F I K A A S C G A L L T<br>F I K A A S C G A L L T G<br>I K A A S C G A L L T G A<br>K A A S C G A L L T G A L<br>A A S C G A L L T G A L P<br>A S C G A L L T G A L P S<br>S C G A L L T G A L P S V<br>C G A L L T G A L P S V S<br>G A L L T G A L P S V S H<br>A L L T G A L P S V S H A<br>L L T G A L P S V S H A A<br>L T G A L P S V S H A A A<br>T G A L P S V S H A A A E<br>G A L P S V S H A A A E N<br>A L P S V S H A A A E N R<br>L P S V S H A A A E N R P<br>P S V S H A A A E N R P P<br>S V S H A A A E N R P P I	NapG<br>M S R S A K P Q N G R R R<br>R S A K P Q N G R R R F L<br>A K P Q N G R R R F L R D<br>P Q N G R R R F L R D V V<br>N G R R R F L R D V V R T<br>R R R F L R D V V R T A G<br>R F L R D V V R T A G G L<br>L R D V V R T A G G L A A<br>D V V R T A G G L A A V G<br>V R T A G G L A A V G V A<br>T A G G L A A V G V A L G<br>G G L A A V G V A L G L Q<br>L A A V G V A L G L Q Q Q<br>A V G V A L G L Q Q Q T A<br>G V A L G L Q Q Q T A R A<br>A L G L Q Q Q T A R A S G<br>G L Q Q Q T A R A S G V R<br>Q Q Q T A R A S G V R L R
NrfC<br>M T W S R R Q F L T G V G<br>T W S R R Q F L T G V G V<br>W S R R Q F L T G V G V L<br>S R R Q F L T G V G V L A<br>R R Q F L T G V G V L A A<br>R Q F L T G V G V L A A V<br>Q F L T G V G V L A A V S<br>F L T G V G V L A A V S G<br>L T G V G V L A A V S G T<br>T G V G V L A A V S G T A	YagT<br>M S N Q G E Y P E D N R V<br>N Q G E Y P E D N R V G K<br>G E Y P E D N R V G K H E<br>Y P E D N R V G K H E P H<br>E D N R V G K H E P H D L<br>N R V G K H E P H D L S L<br>V G K H E P H D L S L T R<br>K H E P H D L S L T R R D<br>E P H D L S L T R R D L I<br>H D L S L T R R D L I K V

G V G V L A A V S G T A G<br>V G V L A A V S G T A G R<br>G V L A A V S G T A G R V<br>V L A A V S G T A G R V V<br>L A A V S G T A G R V V A<br>A A V S G T A G R V V A K<br>A V S G T A G R V V A K T<br>V S G T A G R V V A K T L<br>S G T A G R V V A K T L N<br>G T A G R V V A K T L N I<br>T A G R V V A K T L N I N	L S L T R R D L I K V S A<br>L T R R D L I K V S A A T<br>R R D L I K V S A A T A A<br>D L I K V S A A T A A T A<br>I K V S A A T A A T A V V<br>V S A A T A A T A V V Y P<br>A A T A A T A V V Y P H S<br>T A A T A V V Y P H S T L<br>A T A V V Y P H S T L A A<br>A V V Y P H S T L A A S V<br>V Y P H S T L A A S V P A<br>P H S T L A A S V P A A T<br>S T L A A S V P A A T P A<br>L A A S V P A A T P A P E
YdhX<br>M S W I G W T V A A T A L<br>W I G W T V A A T A L G D<br>G W T V A A T A L G D N Q<br>T V A A T A L G D N Q M S<br>A A T A L G D N Q M S F T<br>T A L G D N Q M S F T R R<br>L G D N Q M S F T R R K F<br>D N Q M S F T R R K F V L<br>Q M S F T R R K F V L G M<br>S F T R R K F V L G M G T<br>T R R K F V L G M G T V I<br>R K F V L G M G T V I F F<br>F V L G M G T V I F F T G<br>L G M G T V I F F T G S A<br>M G T V I F F T G S A S S<br>T V I F F T G S A S S L L<br>I F F T G S A S S L L A N<br>F T G S A S S L L A N T R<br>G S A S S L L A N T R Q E<br>S A S S L L A N T R Q E K	TorA<br>M N N N D L F Q A S R R R<br>N N D L F Q A S R R R F L<br>D L F Q A S R R R F L A Q<br>F Q A S R R R F L A Q L G<br>A S R R R F L A Q L G G L<br>R R R F L A Q L G G L T V<br>R F L A Q L G G L T V A G<br>L A Q L G G L T V A G M L<br>Q L G G L T V A G M L G P<br>G G L T V A G M L G P S L<br>L T V A G M L G P S L L T<br>V A G M L G P S L L T P R<br>G M L G P S L L T P R R A<br>L G P S L L T P R R A T A<br>P S L L T P R R A T A A Q<br>L L T P R R A T A A Q A A<br>T P R R A T A A Q A A T D<br>R R A T A A Q A A T D A V<br>R A T A A Q A A T D A V I
TorZ<br>M T L T R R E F I K H S G<br>T L T R R E F I K H S G I<br>L T R R E F I K H S G I A<br>T R R E F I K H S G I A A<br>R R E F I K H S G I A A G<br>R E F I K H S G I A A G A<br>E F I K H S G I A A G A L<br>F I K H S G I A A G A L V<br>I K H S G I A A G A L V V<br>K H S G I A A G A L V V T<br>H S G I A A G A L V V T S<br>S G I A A G A L V V T S A<br>G I A A G A L V V T S A A<br>I A A G A L V V T S A A P<br>A A G A L V V T S A A P L<br>A G A L V V T S A A P L P<br>G A L V V T S A A P L P A<br>A L V V T S A A P L P A W<br>L V V T S A A P L P A W A<br>V V T S A A P L P A W A E<br>V T S A A P L P A W A E E<br>T S A A P L P A W A E E K<br>S A A P L P A W A E E K G<br>A A P L P A W A E E K G G<br>A P L P A W A E E K G G K	NapA<br>M K L S R R S F M K A N A<br>K L S R R S F M K A N A V<br>L S R R S F M K A N A V A<br>S R R S F M K A N A V A A<br>R R S F M K A N A V A A A<br>R S F M K A N A V A A A A<br>S F M K A N A V A A A A A<br>F M K A N A V A A A A A A<br>M K A N A V A A A A A A A<br>K A N A V A A A A A A A G<br>A N A V A A A A A A A G L<br>N A V A A A A A A A G L S<br>A V A A A A A A A G L S V<br>V A A A A A A A G L S V P<br>A A A A A A A G L S V P G<br>A A A A A A G L S V P G V<br>A A A A A G L S V P G V A<br>A A A A G L S V P G V A R<br>A A A G L S V P G V A R A<br>A A G L S V P G V A R A V<br>A G L S V P G V A R A V V<br>G L S V P G V A R A V V G<br>L S V P G V A R A V V G Q<br>S V P G V A R A V V G Q Q<br>V P G V A R A V V G Q Q E

DmsA

M K T K I P D A V L A A E
T K I P D A V L A A E V S
I P D A V L A A E V S R R
D A V L A A E V S R R G L
V L A A E V S R R G L V K
A A E V S R R G L V K T T
E V S R R G L V K T T A I
S R R G L V K T T A I G G
R G L V K T T A I G G L A
L V K T T A I G G L A M A
K T T A I G G L A M A S S
T A I G G L A M A S S A L
I G G L A M A S S A L T L
G L A M A S S A L T L P F
A M A S S A L T L P F S R
A S S A L T L P F S R I A
S A L T L P F S R I A H A
L T L P F S R I A H A V D
L P F S R I A H A V D S A
F S R I A H A V D S A I P

YnfE

M S K N E R M V G I S R R
K N E R M V G I S R R T L
E R M V G I S R R T L V K
M V G I S R R T L V K S T
G I S R R T L V K S T A I
S R R T L V K S T A I G S
R T L V K S T A I G S L A
L V K S T A I G S L A L A
K S T A I G S L A L A A G
T A I G S L A L A A G G F
I G S L A L A A G G F S L
S L A L A A G G F S L P F
A L A A G G F S L P F T L
A A G G F S L P F T L R N
G G F S L P F T L R N A A
F S L P F T L R N A A A A
L P F T L R N A A A A V Q
F T L R N A A A A V Q Q A
T L R N A A A A V Q Q A R

YnfF

M M K I H T T E A L M K A
K I H T T E A L M K A E I
H T T E A L M K A E I S R
T E A L M K A E I S R R S
A L M K A E I S R R S L M
M K A E I S R R S L M K T
A E I S R R S L M K T S A
I S R R S L M K T S A L G
R R S L M K T S A L G S L
S L M K T S A L G S L A L
M K T S A L G S L A L A S
T S A L G S L A L A S S A
A L G S L A L A S S A F T
G S L A L A S S A F T L P
L A L A S S A F T L P F S
L A S S A F T L P F S Q M
S S A F T L P F S Q M V R
A F T L P F S Q M V R A A
T L P F S Q M V R A A E A
P F S Q M V R A A E A P V
F S Q M V R A A E A P V E

FdnG

M D V S R R Q F F K I C A
V S R R Q F F K I C A G G
R R Q F F K I C A G G M A
Q F F K I C A G G M A G T
F K I C A G G M A G T T V
I C A G G M A G T T V A A
A G G M A G T T V A A L G
G M A G T T V A A L G F A
A G T T V A A L G F A P K
T T V A A L G F A P K Q A
V A A L G F A P K Q A L A
A L G F A P K Q A L A Q A
G F A P K Q A L A Q A R N
A P K Q A L A Q A R N Y K

FdoG

M Q V S R R Q F F K I C A
V S R R Q F F K I C A G G
R R Q F F K I C A G G M A
Q F F K I C A G G M A G T
F K I C A G G M A G T T A
I C A G G M A G T T A A A
A G G M A G T T A A A L G
G M A G T T A A A L G F A
A G T T A A A L G F A P S
T T A A A L G F A P S V A
A A A L G F A P S V A L A
A L G F A P S V A L A E T
G F A P S V A L A E T R Q
A P S V A L A E T R Q Y K

YedY

M K R R Q V L K A L G I S
K R R Q V L K A L G I S A
R R Q V L K A L G I S A T
R Q V L K A L G I S A T A
Q V L K A L G I S A T A L
V L K A L G I S A T A L S
L K A L G I S A T A L S L
K A L G I S A T A L S L P
A L G I S A T A L S L P H
L G I S A T A L S L P H A
G I S A T A L S L P H A A
I S A T A L S L P H A A H
S A T A L S L P H A A H A
A T A L S L P H A A H A D
T A L S L P H A A H A D L
A L S L P H A A H A D L L
L S L P H A A H A D L L S

	S L P H A A H A D L L S W<br>L P H A A H A D L L S W F
CueO<br>M Q R R D F L K Y S V A L<br>Q R R D F L K Y S V A L G<br>R R D F L K Y S V A L G V<br>R D F L K Y S V A L G V A<br>D F L K Y S V A L G V A S<br>F L K Y S V A L G V A S A<br>L K Y S V A L G V A S A L<br>K Y S V A L G V A S A L P<br>Y S V A L G V A S A L P L<br>S V A L G V A S A L P L W<br>V A L G V A S A L P L W S<br>A L G V A S A L P L W S R<br>L G V A S A L P L W S R A<br>G V A S A L P L W S R A V<br>V A S A L P L W S R A V F<br>A S A L P L W S R A V F A<br>S A L P L W S R A V F A A<br>A L P L W S R A V F A A E<br>L P L W S R A V F A A E R<br>P L W S R A V F A A E R P<br>L W S R A V F A A E R P T<br>W S R A V F A A E R P T L	SufI<br>M S L S R R Q F I Q A S G<br>S L S R R Q F I Q A S G I<br>L S R R Q F I Q A S G I A<br>S R R Q F I Q A S G I A L<br>R R Q F I Q A S G I A L C<br>R Q F I Q A S G I A L C A<br>Q F I Q A S G I A L C A G<br>F I Q A S G I A L C A G A<br>I Q A S G I A L C A G A V<br>Q A S G I A L C A G A V P<br>A S G I A L C A G A V P L<br>S G I A L C A G A V P L K<br>G I A L C A G A V P L K A<br>I A L C A G A V P L K A S<br>A L C A G A V P L K A S A<br>L C A G A V P L K A S A A<br>C A G A V P L K A S A A G<br>A G A V P L K A S A A G Q<br>G A V P L K A S A A G Q Q<br>A V P L K A S A A G Q Q Q<br>V P L K A S A A G Q Q Q P
YahJ<br>M K E S N S R R E F L S Q<br>E S N S R R E F L S Q S G<br>N S R R E F L S Q S G K M<br>R R E F L S Q S G K M V T<br>E F L S Q S G K M V T A A<br>L S Q S G K M V T A A A L<br>Q S G K M V T A A A L F G<br>G K M V T A A A L F G T S<br>M V T A A A L F G T S V P<br>T A A A L F G T S V P L A<br>A A L F G T S V P L A H A<br>L F G T S V P L A H A A V<br>G T S V P L A H A A V A G<br>S V P L A H A A V A G T L	WcaM<br>M P F K K L S R R T F L T<br>P F K K L S R R T F L T A<br>F K K L S R R T F L T A S<br>K K L S R R T F L T A S S<br>K L S R R T F L T A S S A<br>L S R R T F L T A S S A L<br>S R R T F L T A S S A L A<br>R R T F L T A S S A L A F<br>R T F L T A S S A L A F L<br>T F L T A S S A L A F L H<br>F L T A S S A L A F L H T<br>L T A S S A L A F L H T P<br>T A S S A L A F L H T P F<br>A S S A L A F L H T P F A<br>S S A L A F L H T P F A R<br>S A L A F L H T P F A R A<br>A L A F L H T P F A R A L<br>L A F L H T P F A R A L P<br>A F L H T P F A R A L P A<br>F L H T P F A R A L P A R<br>L H T P F A R A L P A R Q<br>H T P F A R A L P A R Q S
MdoD<br>M D R R R F I K G S M A M<br>R R R F I K G S M A M A A<br>R F I K G S M A M A A V C<br>I K G S M A M A A V C G T<br>G S M A M A A V C G T S G<br>M A M A A V C G T S G I A<br>M A A V C G T S G I A S L<br>A V C G T S G I A S L F S<br>C G T S G I A S L F S Q A<br>T S G I A S L F S Q A A F<br>G I A S L F S Q A A F A A	EfeB (= YcdB)<br>M Q Y K D E N G V N E P S<br>Y K D E N G V N E P S R R<br>D E N G V N E P S R R R L<br>N G V N E P S R R R L L K<br>V N E P S R R R L L K V I<br>E P S R R R L L K V I G A<br>S R R R L L K V I G A L A<br>R R L L K V I G A L A L A<br>L L K V I G A L A L A G S<br>K V I G A L A L A G S C P<br>I G A L A L A G S C P V A

A S L F S Q A A F A A D S<br>L F S Q A A F A A D S D I<br>F S Q A A F A A D S D I A	A L A L A G S C P V A H A<br>A L A G S C P V A H A Q K<br>A G S C P V A H A Q K T Q<br>S C P V A H A Q K T Q S A
YaeI<br>M I S R R R F L Q A T A A<br>I S R R R F L Q A T A A T<br>S R R R F L Q A T A A T I<br>R R R F L Q A T A A T I A<br>R R F L Q A T A A T I A T<br>R F L Q A T A A T I A T S<br>F L Q A T A A T I A T S S<br>L Q A T A A T I A T S S G<br>Q A T A A T I A T S S G F<br>A T A A T I A T S S G F G<br>T A A T I A T S S G F G Y<br>A A T I A T S S G F G Y M<br>A T I A T S S G F G Y M H<br>T I A T S S G F G Y M H Y<br>I A T S S G F G Y M H Y C<br>A T S S G F G Y M H Y C E<br>T S S G F G Y M H Y C E P<br>S S G F G Y M H Y C E P G<br>S G F G Y M H Y C E P G W<br>G F G Y M H Y C E P G W F<br>F G Y M H Y C E P G W F E	AmiA<br>M S T F K P L K T L T S R<br>T F K P L K T L T S R R Q<br>K P L K T L T S R R Q V L<br>L K T L T S R R Q V L K A<br>T L T S R R Q V L K A G L<br>T S R R Q V L K A G L A A<br>R R Q V L K A G L A A L T<br>Q V L K A G L A A L T L S<br>L K A G L A A L T L S G M<br>A G L A A L T L S G M S Q<br>L A A L T L S G M S Q A I<br>A L T L S G M S Q A I A K<br>T L S G M S Q A I A K D E<br>S G M S Q A I A K D E L L<br>G M S Q A I A K D E L L K
AmiC<br>M S G S N T A I S R R R L<br>S G S N T A I S R R R L L<br>G S N T A I S R R R L L Q<br>S N T A I S R R R L L Q G<br>N T A I S R R R L L Q G A<br>T A I S R R R L L Q G A G<br>A I S R R R L L Q G A G A<br>I S R R R L L Q G A G A M<br>S R R R L L Q G A G A M W<br>R R R L L Q G A G A M W L<br>R R L L Q G A G A M W L L<br>R L L Q G A G A M W L L S<br>L L Q G A G A M W L L S V<br>L Q G A G A M W L L S V S<br>Q G A G A M W L L S V S Q<br>G A G A M W L L S V S Q V<br>A G A M W L L S V S Q V S<br>G A M W L L S V S Q V S L<br>A M W L L S V S Q V S L A<br>M W L L S V S Q V S L A A<br>W L L S V S Q V S L A A V<br>L L S V S Q V S L A A V S<br>L S V S Q V S L A A V S Q<br>S V S Q V S L A A V S Q V<br>V S Q V S L A A V S Q V V	FhuD<br>M S G L P L I S R R R L L<br>S G L P L I S R R R L L T<br>G L P L I S R R R L L T A<br>L P L I S R R R L L T A M<br>P L I S R R R L L T A M A<br>L I S R R R L L T A M A L<br>I S R R R L L T A M A L S<br>S R R R L L T A M A L S P<br>R R R L L T A M A L S P L<br>R R L L T A M A L S P L L<br>R L L T A M A L S P L L W<br>L L T A M A L S P L L W Q<br>L T A M A L S P L L W Q M<br>T A M A L S P L L W Q M N<br>A M A L S P L L W Q M N T<br>M A L S P L L W Q M N T A<br>A L S P L L W Q M N T A H<br>L S P L L W Q M N T A H A<br>S P L L W Q M N T A H A A<br>P L L W Q M N T A H A A A<br>L L W Q M N T A H A A A I<br>L W Q M N T A H A A A I D<br>W Q M N T A H A A A I D P<br>Q M N T A H A A A I D P N
YcbK<br>M D K F D A N R R K L L A<br>D K F D A N R R K L L A L<br>K F D A N R R K L L A L G<br>F D A N R R K L L A L G G<br>D A N R R K L L A L G G V<br>A N R R K L L A L G G V A<br>N R R K L L A L G G V A L	HiPIP<br>M S D K P I S K S R R D A<br>D K P I S K S R R D A V K<br>P I S K S R R D A V K V M<br>S K S R R D A V K V M L G<br>S R R D A V K V M L G T A<br>R D A V K V M L G T A A A<br>A V K V M L G T A A A I P

R R K L L A L G G V A L G<br>R K L L A L G G V A L G A<br>K L L A L G G V A L G A A<br>L L A L G G V A L G A A I<br>L A L G G V A L G A A I L<br>A L G G V A L G A A I L P<br>L G G V A L G A A I L P T<br>G G V A L G A A I L P T P<br>G V A L G A A I L P T P A<br>V A L G A A I L P T P A F<br>A L G A A I L P T P A F A<br>L G A A I L P T P A F A T<br>G A A I L P T P A F A T L<br>A A I L P T P A F A T L S<br>A I L P T P A F A T L S T<br>I L P T P A F A T L S T P<br>L P T P A F A T L S T P R	K V M L G T A A A I P M I<br>M L G T A A A I P M I N L<br>G T A A A I P M I N L V G<br>A A A I P M I N L V G F G<br>A I P M I N L V G F G T A<br>P M I N L V G F G T A R A<br>I N L V G F G T A R A S A<br>L V G F G T A R A S A P A<br>G F G T A R A S A P A N A
