# Supplementary material for: Positive charges promote the recognition of proteins by the chaperone SlyD from Escherichia coli
Source: PLoS One. 2024 Jun 25;19(6):e0305823. doi: 10.1371/journal.pone.0305823 (PMC11198818; doi:10.1371/journal.pone.0305823)
Supplement: S3 Table — The asterisk (*) marks output data that reflect no specific interaction. (PDF) [file pone.0305823.s007.pdf]

S3 Table. N-values, affinities and enthalpies of SlyD interactions with HiPIP precursor (RR-HiPIP), as derived from ITC. The asterisk (*) marks output data that reflect no specific interaction.

Variant	N	K_D [µM]	H [Cal/mol]
WT	0.710 ± 0.002	0.35 ± 0.02	-9060 ± 37.2
Y68S	0.462 ± 0.003	0.30 ± 0.02	-9560 ± 72.5
E73A	0.616 ± 0.003	0.32 ± 0.02	-11200 ± 77.4
F84A	0.766 ± 0.011	6.10 ± 0.57	-17100 ± 484
F84N	0.532 ± 0.004	6.62 ± 0.20	-31700 ± 443
E89A	0.404 ± 0.004	0.38 ± 0.04	-13500 ± 179
F96A	0.660 ± 0.013	4.88 ± 0.54	-20000 ± 688
F96G	0.575 ± 0.007	5.65 ± 0.30	-29400 ± 600
F96N	0.628 ± 0.036	8.85 ± 2.78	-11600 ± 1520
D101A	0.604 ± 0.012	0.95 ± 0.16	-6890 ± 178
E108A	0.569 ± 0.015	1.82 ± 0.32	-5860 ± 220
E113A	0.616 ± 0.008	0.49 ± 0.07	-15400 ± 266
D115A	0.541 ± 0.026	0.27 ± 0.02	-16000 ± 101
F84N + F96N	*0.352 ± 78.9**	*361 ± 14,600**	*-7980000 ± 20500000**
