# Supplementary material for: Positive charges promote the recognition of proteins by the chaperone SlyD from Escherichia coli
Source: PLoS One. 2024 Jun 25;19(6):e0305823. doi: 10.1371/journal.pone.0305823 (PMC11198818; doi:10.1371/journal.pone.0305823)
Supplement: S1 Raw images — (PDF) [file pone.0305823.s009.pdf]

Fig 4B

left gel: Coomassie-stained SlyD SDS PAGE analysis, original image

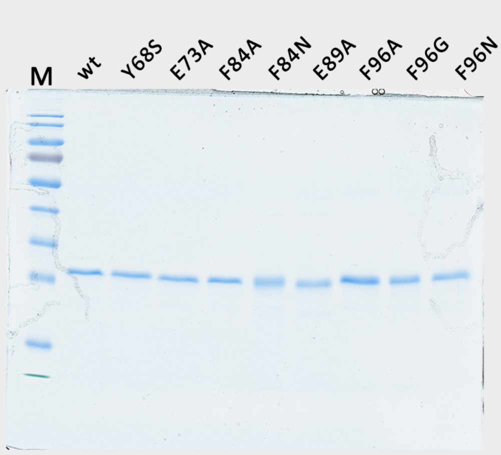

right gel: Coomassie-stained SlyD SDS PAGE analysis, original image; note that lane 6 is unrelated to the study and has not been shown in the manuscript.

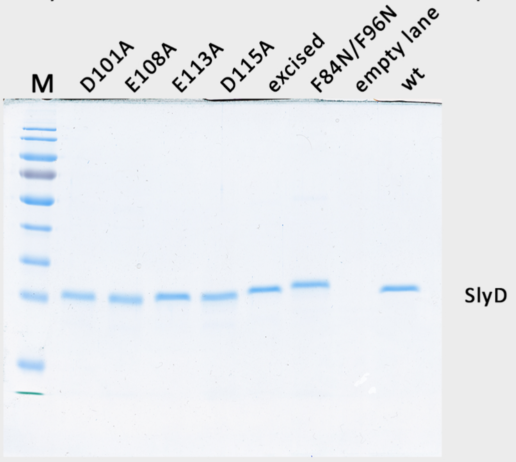

S2 Fig

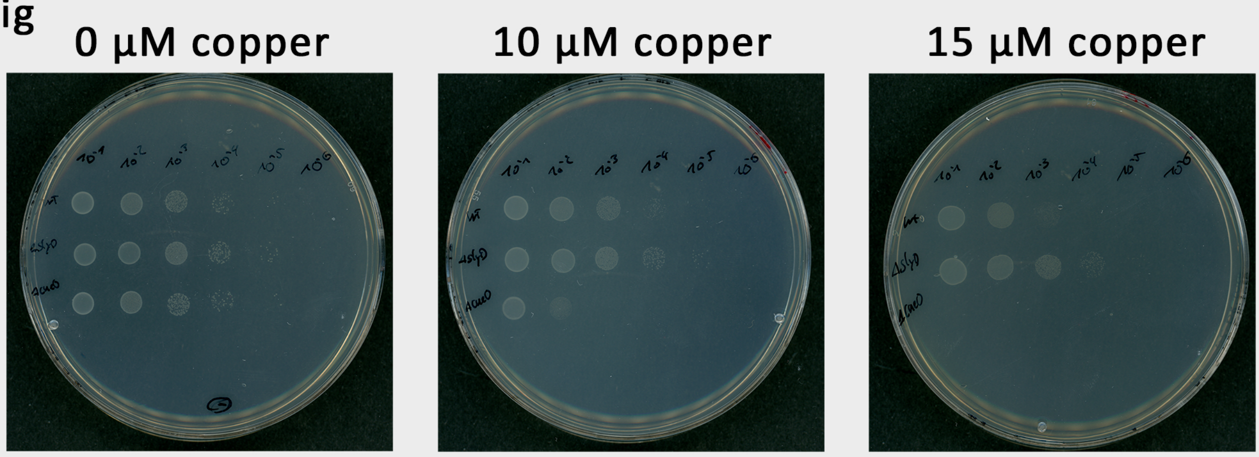

whole agar plate images, original data

S3 Fig

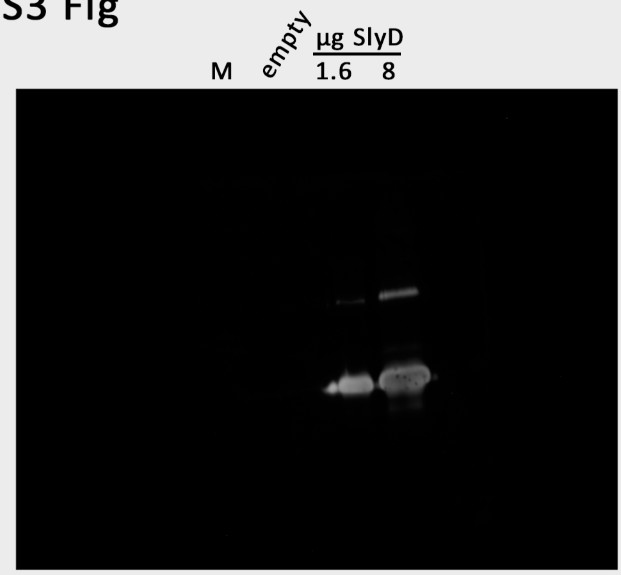

SlyD-Blot - original data, not inverted, not cropped, not rotated

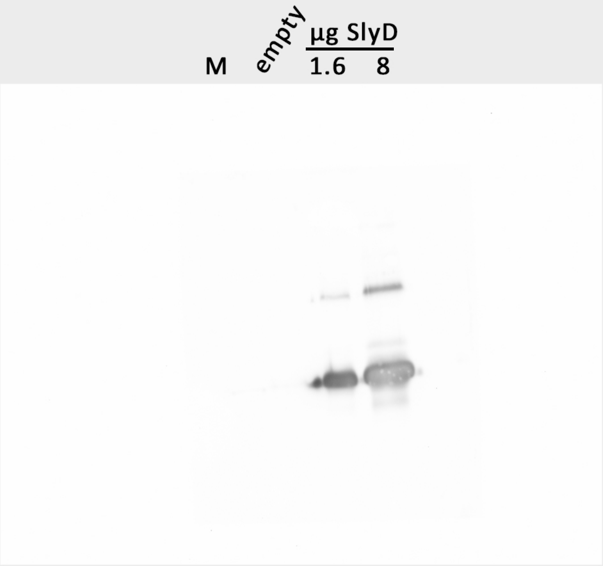

SlyD-Blot - original data, inverted, not cropped, not rotated

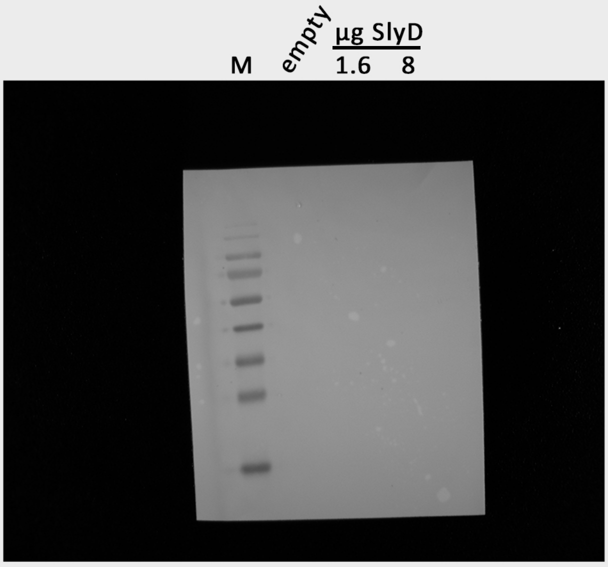

SlyD-Blot - Marker, original data, not cropped, not rotated

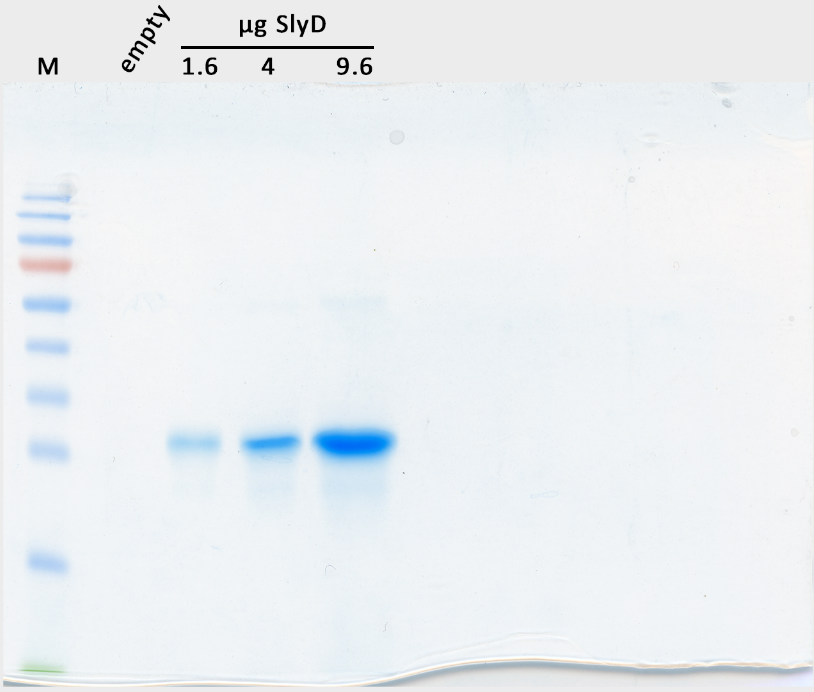

SlyD-Coomassie-stained SDS-PAGE gel, original data, whole gel; only the 9.6 µg SlyD lane is in the Figure
